# The mediating effect of self-esteem on the relationship between perceived discrimination and psychological well-being in immigrants

**DOI:** 10.1371/journal.pone.0198413

**Published:** 2018-06-21

**Authors:** Alfonso Urzúa, Rodrigo Ferrer, Nidia Godoy, Francisca Leppes, Carlos Trujillo, Camila Osorio, Alejandra Caqueo-Urízar

**Affiliations:** 1 Escuela de Psicología, Universidad Católica del Norte, Antofagasta, Chile; 2 Departamento de Filosofía y Psicología, Universidad de Tarapacá, Arica, Chile; Universidad del Desarrollo, CHILE

## Abstract

The aim of the study is to analyze the mediating effect of self-esteem on the relationship between perceived discrimination and psychological well-being in South American immigrants in Chile. An analytical, cross sectional, non-experimental design was used. We evaluated 853 Peruvians and Colombians living in the northern cities of Arica, Antofagasta, and Santiago de Chile, the capital located in the center of the country. The instruments used were the Ryff Psychological Well-being Scale, the Rosenberg Self-Esteem Scale and the Perceived Discrimination Scale by Basabe, Paez, Aierdi and Jiménez-Aristizabal. We used the estimation method (RWLS) and polychoric correlation matrices, to estimate the effect size and overall fit of the direct effect models of discrimination and self-esteem on psychological well-being, and indirect and total effects of discrimination mediated by self-esteem. While both populations reported similar levels of perceived discrimination, it was found that the means in psychological well-being and self-esteem of the Colombian population were significantly higher than that of the Peruvian population. Regarding self-esteem, the results provided evidence for the possible mediating effect on the relationship between perceived discrimination and psychological well-being. This research aims to contribute to the development of interventions seeking to strengthen self-esteem in order to circumvent possible negative consequences of perceived discrimination, as a consequent, improving immigrants´ personal resources to successfully cope with the diverse demands of their new context.

## Introduction

Migration can be understood as the movement of people from one country or region to another to improve personal social and material conditions, and that of their families [1].

Due to various economic and for reasons of security, Chile has become an attractive destination for individuals and families looking for opportunities and conditions which do not exist in their homeland [2,3] consequently, increasing the flow of immigrants to the country. In the 2002 census, immigrants constituted 1.22% of the total population [4], while in 2014 they reached 2.3% of the national population, of which 73% are from South American origins [5].

Migration mainly arises from the discrepancy between the possibilities offered by the migrant´s homeland and the aspirations and expectations of migrants [6]. The reality faced by immigrants in their new residence often contrasts with their expectations. Often this process leads to negative consequences, from physical and mental health problems to economical ones, such as unemployment, marginalization, invisibility, exploitation and discrimination; and variables that directly influence their quality of life and well-being [7].

These numerous problems imply that migration itself is a stressful activity, as moving from one location to another, means being exposed to different environmental conditions. This situation sometimes means living in overcrowded environments, being potential victims of sexual exploitation and other types of violence [8]. These problems can affect their behavior, their social relationships and their general health [9]. It can also diminish immigrants´ quality of life [3, 10], mental health [11–12]), social wellbeing [13], self-reported health [14], and generate distress [15], among others.

One of the areas in which the impact of migration has been seen, is in the area of psychological well-being (PW). This is linked to human development, where a person’s life acquires meaning, to prevail over and achieve valuable goals. A person’s central task is to recognize and realize their full potential, emphasizing that a person’s responsibility is to find the meaning of their existence, even in the face of adversity [16]. There is evidence that PW is not only associated with greater psychological satisfaction, but it also has important implications on physical health [17].

Research on PW has shown links to different factors in immigrant populations such as acculturation strategies, gender, education level, duration of residency, age, marital status, social support, linguistic affinities, income, legal status or labor situations, integration and feeling of belonging in the community or social participation, which may favor or hinder the migration process [18–31]. In addition, other factors influencing immigration may affect adaptation and well-being such as the immigrants´ ethnicity, language, religion or physical appearance [32], these elements could generate integration difficulties triggering a phenomenon such as discrimination and segregation by the host country [33].

Discrimination is conceptualized as the difference in treatment towards a group with common characteristics or towards a person belonging to that group [34]. As a result, a phenomenon called perceived discrimination arises, which refers to the experience experienced by a person where the person feels victimized by discrimination, this constitutes a cognitive process inserted into a socio-cultural and historical context which manifests itself via discourse [35].

The negative influence of perceived discrimination on the physical and mental health of people belonging to various stigmatized groups has been widely reported [36]. People who are perceived as more discriminated against have higher levels of sensitivity, feel worthlessness, guilt, sadness, hopelessness and helplessness [37], do not participate in healthy behaviors, and show disinterest in participating in social and health networks [38]. In addition, they exhibit higher levels of depression, anxiety and psychological stress [39–44], lower well-being [45–46], attenuating a positive relationship between linguistic competencies and satisfaction with life [47].

In immigrant populations, two of the most studied types of discrimination are ethnicity and race, both relate to the presence of depressive symptoms [48–50], low self-esteem [51], poorer self-reported health [52–53] and mental health [54], as well as lower perceived well-being [40–41, 55–56]

Even though there is evidence of a relationship between discrimination, health and well-being, studies of the factors that may moderate or mediate this relationship are still scarce. Studies show that factors such as race, gender, and sexual orientation, moderate the relationship between perceived discrimination and psychological well-being. In addition, a social support network would be a factor that diminishes the negative effects of stress on PW [57–58] since the presence of social networks such as friends and family, would foster the adaptation of said factors that negatively affect PW [44].

Additionally, lines of research have been developed whose objectives were to analyze the role of self-esteem (SE) in both discrimination and well-being. This is defined as overall self-assessment, plus the feelings that accompany that evaluation [59]. Self-esteem is the feeling that one has for oneself, which can be positive or negative, and is constructed through the evaluation of one’s own personal characteristics [60]. This being a resource that mobilizes individuals allowing them to cushion negative experiences [61] which may affect relationships with others, reflecting on the strengths to face undesirable events and influencing well-being in one way or another[62].

It has been reported that SE is negatively affected by discrimination [39, 43, 51]. On the other hand, there is a positive relationship between SE and PW, since to a large extent the well-being of a person is influenced by the way in which PW is perceived and valued in itself. As a result, modulating aspects of the individual´s life, their family and social interactions [63], become a protective factor in their mental health [64].

As to the possible mediating role of SE, which has been found to be a psychological resource in the adolescent population, mediating the relationship between family variables and problems of criminal behavior [65], and the effect of racial discrimination [66]. At the collective level, SE could attenuate the relationship between the perception of ethnic discrimination, as well as the sense of paranoia in possible discriminatory events [67]. In this scenario, it seems reasonable to think that discrimination perceived by immigrants will affect their psychological well-being, but that this effect may be mediated or moderated by self-esteem (the literature is not conclusive). Therefore, the objective of this research was to analyze the mediating and moderating effect of SE in the relationship between perceived discrimination and psychological well-being. We hypothesize that the best model is self-esteem which has a mediating effect, explaining part of the impact of discrimination on PW.

## Materials and method

### Design and participants

This research is an analytical, non-experimental, cross-sectional study. Given the lack of knowledge of the exact number of the target population, and the characteristics of the minority population, the sampling was non-probabilistic and of an intentional type, performed mainly through the snowball technique, [68] in combination with a system of equitable quotas by *sex*, *country of origin* (Peru, Colombia), *legal status in Chile* (legal, illegal) and by *city of residence* (Arica, Antofagasta, Santiago), following the recommendations for accessing difficult groups [69–71]. The inclusion criteria of the this investigation were: adult male or female, immigrants with Colombian or Peruvian nationality, in addition to having resided for at least 6 months in the cities of Arica, Antofagasta or Santiago de Chile. Participants were mainly surveyed in public institutions such as the Chilean Catholic Migration Institute (INCAMI), the Jesuit Migrants Service, the Immigration and Migration Department, the Consulate of Colombia and Peru, health centers, among others.

The final sample consisted of a total of 853 migrants, characterized by: the mean age of 33.2 years (SD = 9.5 years); 49% (n = 418) men and 51% (n = 433) women; 48.3% (n = 412) Peruvian citizens and 51.7% (n = 441) Colombian citizens; 66.4% (n = 562) immigrants with legal status and 33.6% (n = 284) with illegal status; 24.8% (n = 212) residents of Arica, 50.6% (n = 431) residents of Antofagasta and 24.6% (n = 210) residents of Santiago. There were no interactions (p<0.05) between these categories, except for the city of residence and legal status in Chile (χ2GL = 2 = 90.8; p < .05), given that in Antofagasta the percentage of immigrants 81.6% (n = 351) versus 18.4% (n = 79) are illegal, while in Arica and Santiago the hypothesis of equality of proportions (p>05) was maintained.

### Instruments

Ryff’s Psychological Well-Being Scale [16], adapted to Spanish by Díaz et al [72] was used. This scale has 29 items grouped in 6 dimensions: self-acceptance, positive relationships, autonomy, domain of the environment, purpose in life and personal growth. The response format used was a Likert-type, with scores ranging from 1 to 6, where 1 = *totally disagree* and 6 = *totally agree*. This version has reported evidence of reliability and validity based on the internal structure of the measurement instrument [73–75].

Rosenberg’s Self-Esteem Scale [60] is composed of 10 statements which inquired about the person’s feelings toward himself, with a Likert-type response with scores between 1 and 4, depending on the degree of agreement with the statements. The theoretical values fluctuate between 10 (low self-esteem) and 40 (high self-esteem). The Rosenberg Self-Esteem Scale has reported evidence of validity in Chile and an estimated reliability of 0.75 [76].

Another instrument used was Basabe´s et al. [77], Perceived Discrimination Scale consisting of 5 items that allow the person to evaluate the frequency with which he/she considers themselves as being treated unfairly or negatively given their status as an immigrant, with a Likert scale between 1 (*never*) and 5 (*almost always*). This scale has been used with South American immigrants, reporting reliabilities of between .87 and .88 [18, 56, 78].

### Procedures

This research is part of the FONDECYT Project, Grant No. 1140843, entitled "Factors related to the well-being and quality of life perceived in Latin American immigrants in the North of Chile", which was reviewed and approved by the ethics committees of the National Science Commission and Technology of Chile -CONICYT and the Universidad Católica del Norte. The decision to participate was voluntary and was supported by the signing a consent form.

#### Statistical analysis

First, variables and groups were described through descriptive analyzes and comparisons of means with the IBM SPSS Statistics program, version 21. Subsequently, measurement models were tested and refined, estimating reliabilities by omega coefficients with the 7.4 version of the MPLUS program [79] and, following recommendations of several authors on the factorial analysis of ordinal variables [80], confirming factorial analyzes from the polychoric variables matrix, using the *robust weighted least squares* (RWLS) estimation method, which is robust with non-normal discrete variables [81]. In addition, metric and scalar invariance analyzes were performed according to the country of origin of the migrants (Peru, Colombia), according to the invariance test procedure implemented in MPLUS [82].

Finally, we tested two models of only direct effects, Perceived Discrimination over Psychological Well Being (model 1) and Self-Esteem over Psychological Well Being (model 2); and two alternative models of the role of Self-Esteem in the relation between Perceived Discrimination and Psychological Well Being: a moderation model, according to the propose of Stride, Gardner, Catley & Thomas [83] to test moderation models with latent variables in MPLUS; and a mediational model, with RWLS estimation method, polychoric correlation matrices, and a bootstrap estimation of indirect effects, according to the recommendations Lau and Cheung [84]. All models were analyzed with the 7.4 version of the MPLUS program.

## Results

### Psychological well-being

Table 1 shows the means of the PW domains. As can be seen, with the exception of the domain of positive relations, the means of the Colombian population are higher than the Peruvian population evaluated, being significantly higher in the domains of self-acceptance (t _(828)_ = 4,101, p = 0.000), (t _(825)_ = 3.898, p = 0.000), and in the case of environment domain (t _(823)_ = 2,982, p = 0.003), personal growth (t _(835)_ = 3.576, p = 0,000), purpose in life (t_(825)_ = 3,898;p = 0,000), and in the total score (t _(766)_ = 3.594, p = 0.000).

**Table 1:** Averages and standard deviations of psychological well-being domains by country of origin.

Variable	Columbian Population *M (SD)*	Peruvian Population *M (SD)*
Self-acceptance	5,06 (,96) *	4,78 (1,02)
Positive relationships	3,44 (,55)	3,46 (,56)
Autonomy	3,74 (,72)	3,66 (,70)
Environment domain	4,40 (,86) *	4,22 (,85)
Personal growth	4,80 (,96) *	4,56 (,93)
Purpose in life	5,02 (,94) *	4,76 (,97)
Total score Psychological well-being	4,41 (,59) *	4,25 (,59)

N = 853.

*p < .05.

There were no significant differences between the means reported in the Colombian population (M = 2.31, SD = 1.11) and the Peruvian population evaluated (M = 2.20, SD = 0.99).

#### Individual self-esteem

The individual Colombian population mean in self-esteem (X = 23.29; SD = 3.49) is significantly higher than that of the Peruvian population (X = 21.68; SD = 3.60) (t _(815)_ = 6.464, p = 0.000).

### Measurement models

Table 2 presents the adjustment of the average models of the three scales used. However, some relationships between observations were insufficiently represented by the initial models, with adjustment levels lower than the standards recommended in the literature i.e. CFI> 95, TLI> 95, RMSEA <, 08 [85]. Due to this, we proceeded to iteratively debug the initial models, reducing the scales used for the valuation of *perceived discrimination*, *self-esteem*^*1*^ and *psychological well-being*^*2*^.

**Table 2:** Indicators of global adjustment of measurement models in the total sample.

Measurement model	N° Par	χ^2^	d.f	p	CFI	TLI	RMSEA	RMSEA CI 90%		WRMR
								Low	Upp	
Perceived Discrimination	32	118.796	28	.000	.990	.993	.087	.071	.104	1.551
Perceived Discrimination*	20	15.135	2	.000	.997	.992	.088	.048	.123	.477
Self esteem	28	146.346	13**	.000	.981	.969	.110	.094	.124	1.487
Self esteem*	16	9.943	2	.007	.998	.995	.069	.031	.114	.453
Psychological well-being	189	4299.693	362	.000	.848	.830	.113	.110	.116	2.763
Psychological well-being *	141	977.148	174	.000	.963	.955	.074	.069	.078	1.355

* Debugged model.

** Includes a common method variance factor, to control the effect of the inverted items.

To assess whether the measurement models adequately represent both groups of immigrants, we proceeded to obtain evidence of validity based on intergroup stability from contrasts of factorial invariance (Table 3).

**Table 3:** Contrast of invariance of measurement models between Peruvians and Colombians.

		χ^2^	d.f.	p
Discrimination	Scalar against Configural	27.694	14	0.016
	Metric against Configural	4.281	3	0.233
Self-esteem	Scalar against Configural	12.102	10	0.278
	Metric against Configural	9.099	3	0.028
Psychological well-being	Scalar against Configural	99.698	93	0.299
	Metric against Configural	8.938	15	0.881

We observed that, except for the *self-esteem* variable, metric invariance can be maintained with all scales. However, there are variations among immigrant groups in the metric model.

Finally, the estimates of reliability of each instrument and sub-dimension are presented from the debugged models (Table 4).

**Table 4:** Omega coefficient of dimensions.

	Psychological well-being						
Perceived Discrimination (items = 4)	Self-esteem (items = 4)	Self-acceptance (items = 4)	Positive relationships (items = 4)	Environmental domain (items = 3)	Positive growth (items = 3)	Autonomy (items = 4)	Purpose (items = 5)
.942	.928	.856	.731	.650	.885	.705	.858

It was observed that most of the dimensions studied reported reliability estimates that present adequate levels of internal consistency (ω >.80) or, at least, sufficient (ω >.80), except for the *environment domain dimension*, which seems to be slightly below expected.

### Models of structural equations—Relationships between variables

**Fig 1:**
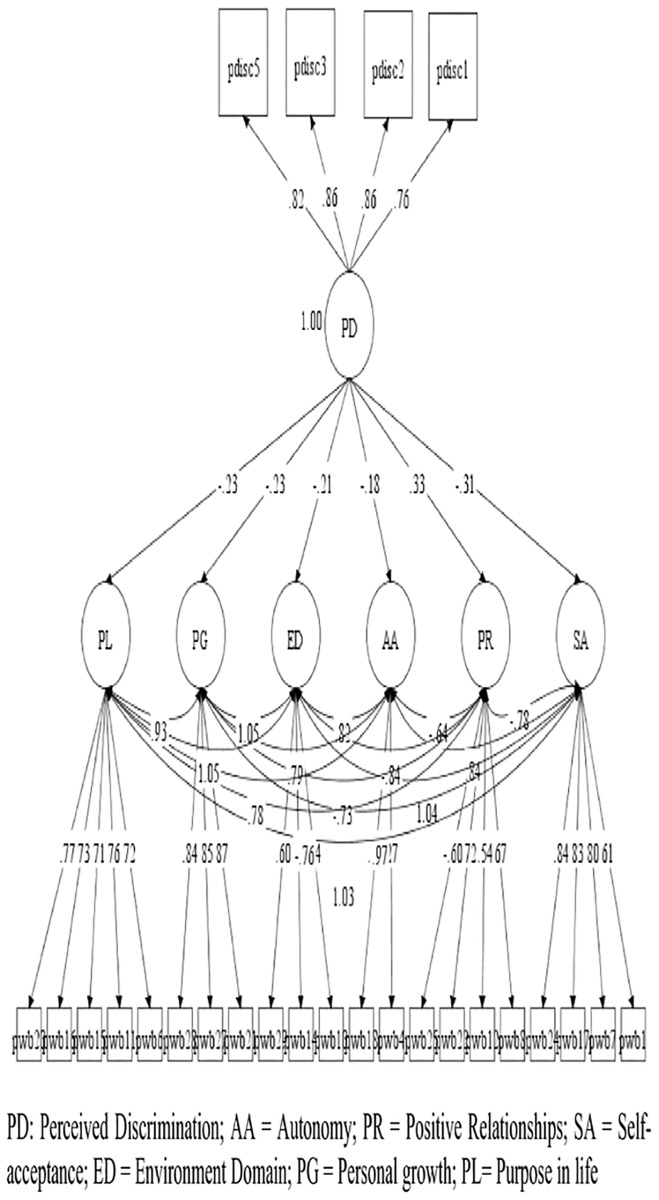
Model of the direct effect of perceived discrimination on psychological well-being. *only relations with p < .05 are represented.

**Fig 2:**
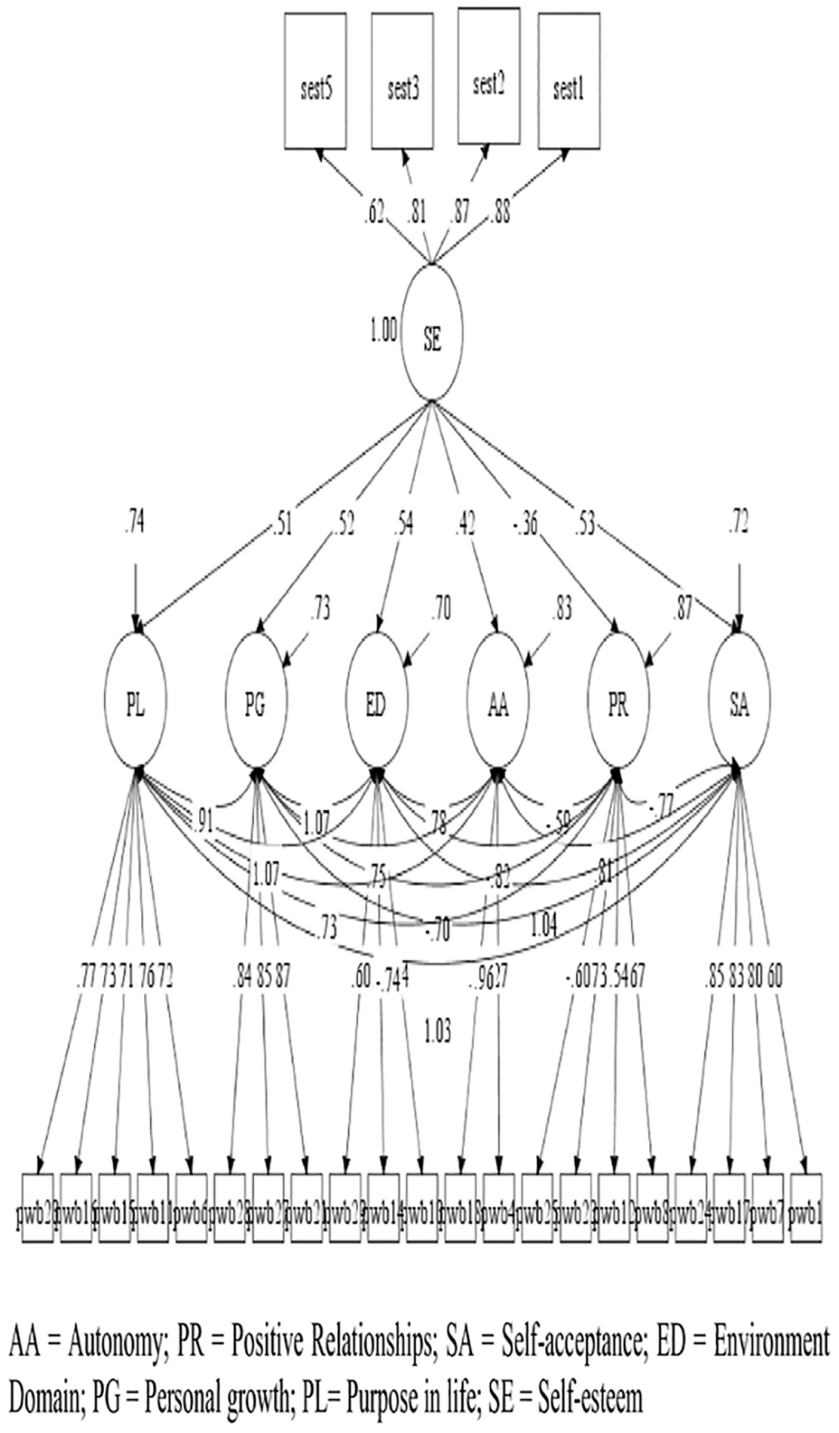
Direct effect of self-esteem on psychological well-being. *only relations with p < .05 are represented.

**Fig 3:**
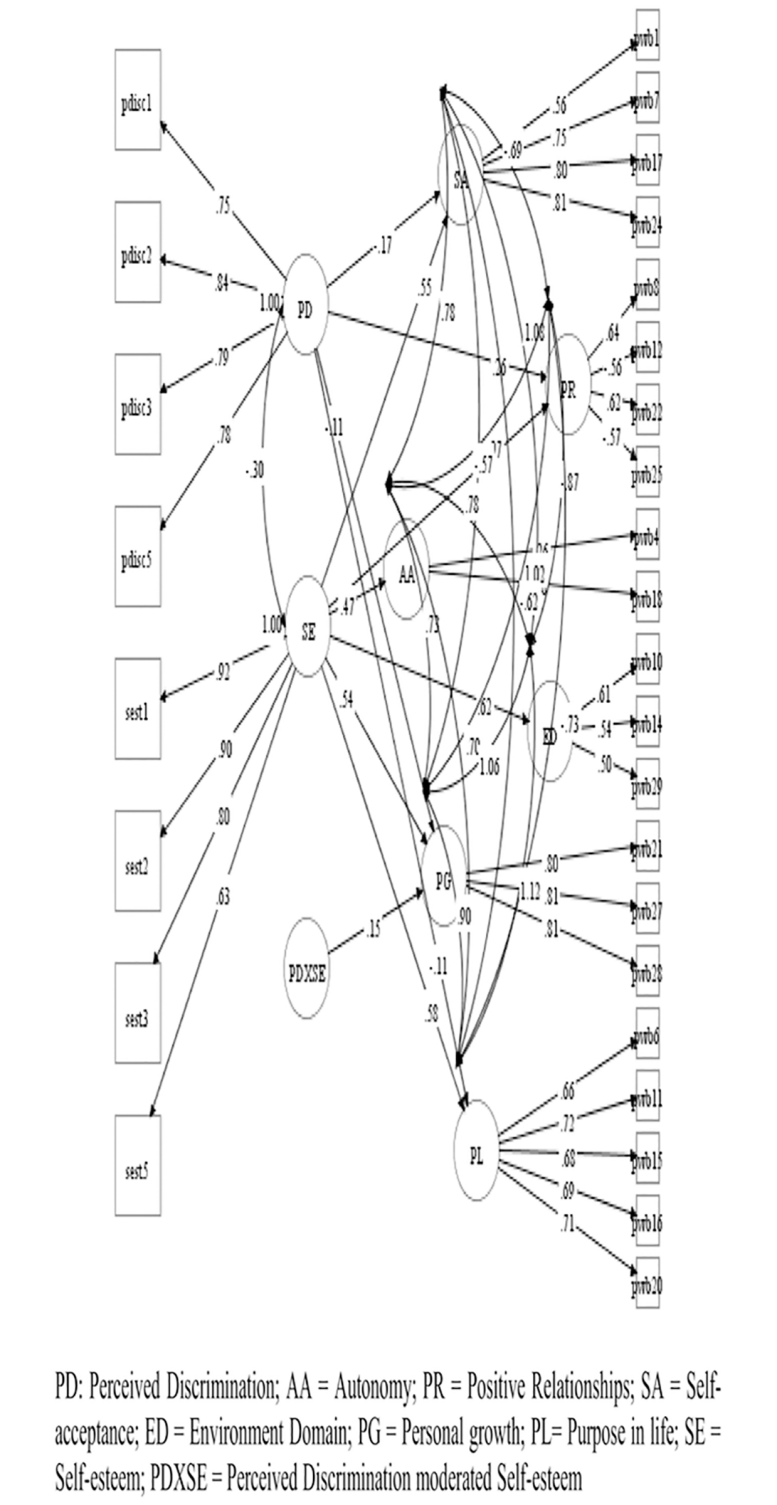
Model with moderated effects of self-esteem, over the relation of perceived discrimination with psychological well-being. *only relations with p < .05 are represented.

**Fig 4:**
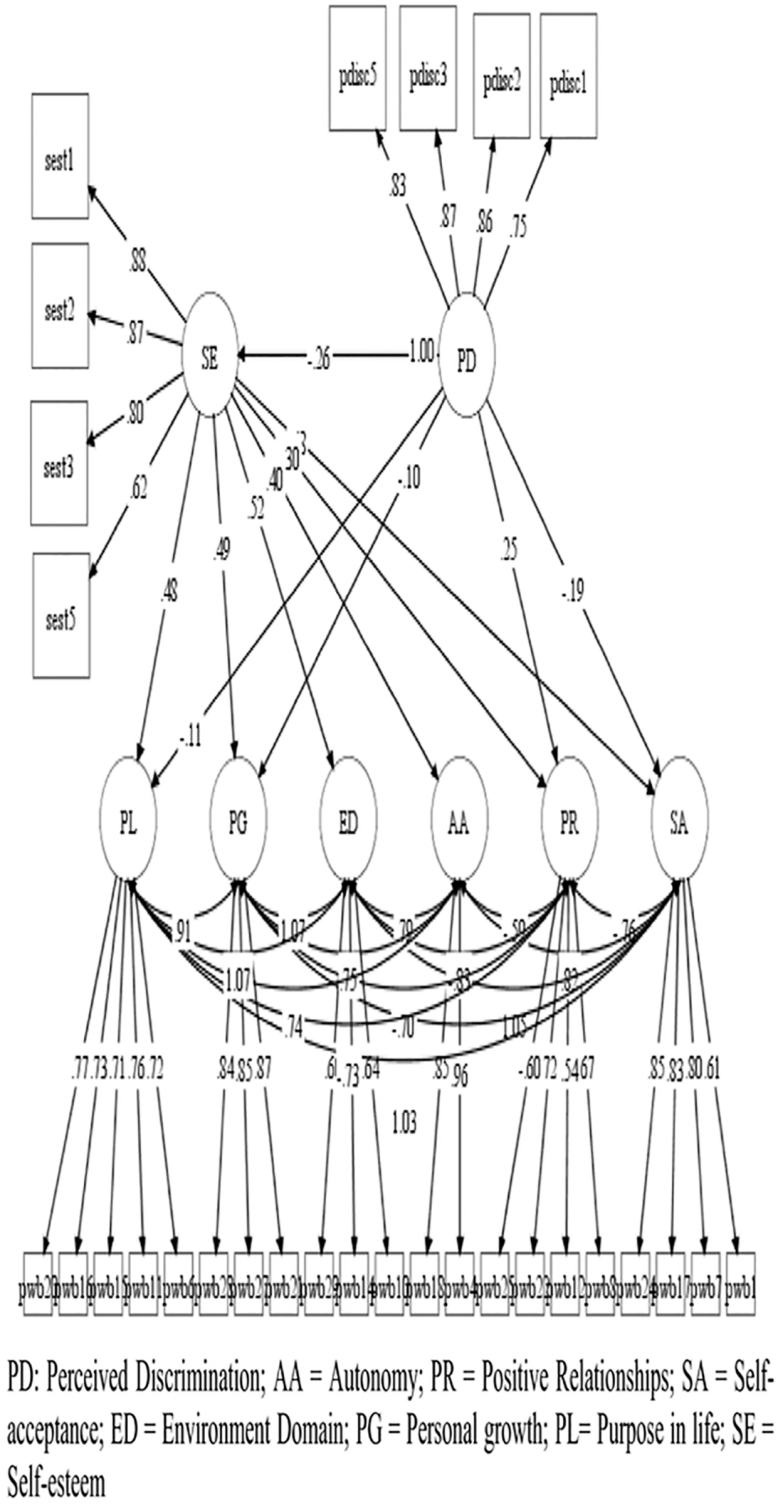
Model with the effects of perceived discrimination, mediated by self-esteem, on psychological well-being. *only relations with p < .05 are represented.

Based on the models of depured measures, four hypothesized models were contrasted in the study: A model of the direct effect of *perceived discrimination* on *psychological well-being* (Fig 1); Another of the direct effect of *self-esteem* on *psychological well-being* (Fig 2); A model with moderated effects of *self-esteem*, *over the relation of perceived discrimination* with *psychological well-being* (Fig 3); And a model with the effects of *perceived discrimination*, mediated by *self-esteem*, on *psychological well-being* (Fig 4).

In models 1 and 2, we observed that *perceived discrimination* presented moderate inverse effects [86] (b>.30) or mild (b>.10) on the different aspects of *psychological well-being*, while *self-esteem* presented mostly large effects (b> .50). In the case of the moderation model (model 3), we only observed a mild effect of moderation of *self-esteem* in the relation between *perceived discrimination* and the dimension *positive growth* of *psychological well-being*. Finally, in the case of the mediated model, we observed that most direct effects of *perceived discrimination* over *psychological well-being dimensions* diminished or disappeared, suggesting a partial moderation of self-esteem in all *psychological well-being dimensions*.

The three models presented adequate adjustment levels (Table 5), are all a good representation of the observed relationships. The moderated model did not provide standard comparable fit statistics. Loglikelihood(H0) = -35201.380; AIC = 70642.760; BIC = 71212.611.

**Table 5:** Global adjustment indicators of the structural models.

Model	N° Par	χ^2^	d.f.	p	CFI	TLI	RMSEA	RMSEA CI		WRMR
								Low	Upp	
Discrimination (Fig 1)	167	1003.080	254	.000	.970	.965	.059	.055	.063	1.328
Self-esteem (Fig 2)	163	931.249	254	.000	.973	.968	.056	.052	.060	1.248
Mediated (Fig 4)	190	1069.479	349	.000	.975	.972	.049	.046	.053	1.242

The standardized estimates of direct effects are presented in Table 6, while the indirect and total effects of discrimination on *psychological well-being*, mediated by *self-esteem*, are presented in Table 7.

**Table 6:** Estimates of standardized effects.

Model		Psychological well-being						Discrimination/Self-esteem
		Self acceptance	Positive relationships	Environmental domain	Positive growth	Autonomy	Purposes	
1	Discrimination	-.309**	.326**	-.212**	-.228**	-.179**	-.230**	-
2	SE	.526**	-.362**	.543**	.515**	.416**	.512**	
3	Discrimination	-.187**	-.250**	-.078	-.103*	-.079	-.106*	-.256**
	SE	.478**	.298**	.523**	.488**	.399**	.485**	

*p < .05.

**p < .01.

**Table 7:** Standardized estimates of indirect and total effects of discrimination on the dimensions of well-being.

Effects	Psychological well-being					
	Self acceptance	Positive relationships	Environmental domain	Positive growth	Autonomy	Purposes
Indirect	-0.122**	0.076**	-0.134**	-0.125**	-0.102**	-0.124**
Total	-0.309**	0.326**	-0.212**	-0.228**	-0.181**	0.230**

**p < .01.

We observed that, when *self-esteem* is incorporated as a mediating variable, an important part of the effects of *perceived discrimination* on the dimensions of *psychological well-being* can be explained from the mediation of self-esteem.

## Discussion

Although numerous studies have repeatedly supported that perceived discrimination is associated with lower self-esteem and negative feelings towards oneself [36, 44, 87], in this research self-esteem was considered as a variable that could have an effect on the relationship between perceived discrimination and psychological well-being. The results support the assumption that self-esteem is a partial mediating variable in this relationship, with the ability to mute the effects of perceived discrimination on the psychological well-being of the participants.

Some of the aspects that could explain this would be that those with a high level of self-esteem would have a positive concept of self. A reason why may be that, when faced with situations of discrimination they would be less affected, since they would not internalize those unfavorable dealings. In accordance with the above, people with a low level of self-esteem would be more susceptible to negative feedback, since this is consistent with their perception of themselves. Furthermore, the presence of low self-esteem could increase uncertainty in self-definition [88]. This is important since the target society often reflects certain negative images about themselves, especially when it comes to certain groups of which negative stereotypes lie [89]. We consider that the subject constructs his/her self-concept from the context in which he/she is situated, and from belonging to a group with a strong social identity, would result in the improvement of individual and collective self-esteem [90].

The results did not show significant differences between Colombians and Peruvians in terms of perceived discrimination. It is possible to think that, although both groups perceive discrimination, they would have different tools to deal with it, which in this case would be explained due to the levels of individual self-esteem possessed by each member of the group. A low or high self-esteem would affect a relationship with others and would be reflected in the ability to face undesirable events, having an influence one way or another, on well-being [62].

Although research has shown that when discrimination focuses at an ethnic origin and culture, it may lead to socio-affective difficulties, creating multiple forms of racial victimization based on this perceived discrimination [91]. It has been reported that ethnic minorities sometimes face situations of discrimination and social exclusion, generating responses where they have to defend themselves, resist and cope with oppression according to their personal values and culture [92]. In this context, individual self-esteem may be one of those resistance responses, especially if group identification responses are generated as a defense mechanism, which strengthens group self-esteem and which in turn affects individual self-esteem. It has been found that there are members of stigmatized groups who, when they recognize that they are victims of discrimination, can increase identification with their ethnic group as part of their coping strategies, counteracting the negative impact that ethnic discrimination has on self-value and on individual self-esteem. Thus, the type of relationship between discrimination and self-esteem will depend on a number of factors, confirming that sometimes discrimination towards a minority group will strengthen ethnic identity, thereby increasing the self-esteem of its members. Nonetheless, discrimination could also lead to marginalization, which could be associated with a low level of self-esteem [93].

These results would allow guidance for diverse interventions, facilitating the construction of psychological programs whose interventions would focus mainly on working at a collective level to strengthen self-esteem, thereby avoiding the negative consequences of discrimination towards the immigrant group. Hence, strengthening a sense of collective identity in itself will generate a positive effect in individual identity and therefore increase individual and collective self-esteem, which could reduce discrimination exhibited towards these groups.

In this sense, interventions should be focused on generating agency and resistance when facing situations of exclusion, and promoting more effective social inclusion devices to not view immigrants as vulnerable victims who require social intervention [94].

Although it was observed that self-esteem has a partial mediating effect on the relationship between psychological well-being and perceived discrimination, it is uncertain whether this effect would change when investigated in other Latin American or Anglo-Saxon immigrant populations. Despite this, the results open new lines of research related to how self-esteem can affect the discrimination/health relationship and how individual self-esteem is built into the immigrant population.

These findings can be a key input in reducing the negative effects of immigrating, mainly due to the need to adapt and respond to the environmental demands of the new context, via available resources that are generally scarce.

## Supporting information

S1 Data MPLUS codes used for analyses the raw data. (DOCX)

S2 Data Raw data file used to conduce the analyses. (DOCX)
