# Supplementary material for: The mediating effect of self-esteem on the relationship between perceived discrimination and psychological well-being in immigrants
Source: PLoS One. 2018 Jun 21;13(6):e0198413. doi: 10.1371/journal.pone.0198413 (PMC6013095; doi:10.1371/journal.pone.0198413)
Supplement: S1 Data — (DOCX) [file pone.0198413.s001.docx]

- 1. MPLUS codes used for analyses the raw data:

**MMPDISC_MM.inp**

DATA:

FILE IS "datitems99.DAT";

FORMAT IS FREE;

TYPE IS INDIVIDUAL;

NOBSERVATIONS ARE 853;

VARIABLE:

NAMES ARE cv1 cv2 cv3 cv4 cv5 cv6 cv7 cv8 cv9 cv10 cv11 cv12 cv13 cv14

cv15 cv16 cv17 cv18 cv19 cv20 cv21 cv22 cv23 cv24 cv25 cv26

sf1 sf2 sf3 sf4 sf5 sf6 sf7 sf8 sf9 sf10 sf11 sf12

GHQ121 GHQ122 GHQ123 GHQ124 GHQ125 GHQ126 GHQ127 GHQ128

GHQ129 GHQ1210 GHQ1211 GHQ1212

di1 di2 di3 di4 di5

keyes1 keyes2 keyes3 keyes4 keyes5 keyes6 keyes7 keyes8 keyes9 keyes10

keyes11 keyes12

keyes13 keyes14 keyes15 keyes16 keyes17 PWB1 PWB2 PWB3 PWB4 PWB5

PWB6 PWB7 PWB8

PWB9 PWB10 PWB11 PWB12 PWB13 PWB14 PWB15 PWB16 PWB17 PWB18

PWB19 PWB20 PWB21

PWB22 PWB23 PWB24 PWB25 PWB26 PWB27 PWB28 PWB29

ruiz1 ruiz2 ruiz3 ruiz4 ruiz5 ruiz6 ruiz7 ruiz8 ruiz9 ruiz10 ruiz11

ruiz12 ruiz13 ruiz14 ruiz15

ruiz16 ruiz17 ruiz18 ruiz19 ruiz20 ruiz21 ruiz22 ruiz23 ruiz24

oq1 oq2 oq3 oq4 oq5 oq6 oq7 oq8 oq9 oq10 oq11 oq12 oq13 oq14 oq15 oq16

oq17 oq18 oq19 oq20

oq21 oq22 oq23 oq24 oq25 oq26 oq27 oq28 oq29 oq30 oq31 oq32 oq33 oq34

oq35 oq36 oq37 oq38 oq39

oq40 oq41 oq42 oq43 oq44 oq45

DSE1 DSE2 DSE3 DSE4 DSE5 DSE6 DSE7 DSE8 DSE9 DSE10

V181 V182

V183 V184 V185 V186

AUTCOL1 AUTCOL2 AUTCOL3 AUTCOL4 AUTCOL5 AUTCOL6

AUTOSIM

V194A V194B V194C

V195A V195B V195C

V196A V196B V196C V196D

V197A V197B V197C V197D V197E V197F

V198A V198B V198C V198D V198E V198F V198G V198H V198I V198J V198K V198L

V199 V200

PDISC1 PDISC2 PDISC3 PDISC4 PDISC5

V206 V207

V208 V209A

V209B V209C V209D V209E V209F V209G V210 V211A1 V211A2 V211B1 V211B2 V211C1

V211C2 V211D1 V211D2

V211E1 V211E2 V211F1 V211F2 V211G1 V211G2 V212 V213 V214 V215 V216 V217 V218

V219 V220 V221 V222

V223 V224

V225 V226 V227 V228 V229 V230 V231 V232 V233 V234 V235 V236 V237 V238_1

V238_2 V239 V240 V241 V242

V243 V244 V245 V246 V247 V248 V249 V250 V251 V252 V253 V254 V255 V256 V257

V258 V259 V260

V261 V262 V263 V264 V265 V266 V267 V268 V269 V270 V271 V272 V273 V274 V275

V276 V277 V278 V279 V280

V281 V282 V283 V284 V285 V286 V287 V288 V289 V290 V291 V292 V293A

V294 V295 V296 V297 V298 V299 V300 V301 V302 V303 V304 V305 V306 V307 V308

V309 V310 V311

V312A V313 V314 V317

V318 V319 V320 V321 V322 V323 V324 V325 V326 V327

SEST1 SEST2 SEST3 SEST4 SEST5 SEST6 SEST7

V335 V336 V337 V338 V339 V340 V341 V342 V343 V344 V345 V346 V347 V348 V349 V350

V351 V352 V353 V354 V355 V356A V356B V356C V356D

V356E V356F V356G V356H V356I V356J V360A V360B V362A V363 V365 V367 V369A

V369B V370B FENOTIPO

CIUDAD PAIS SEXO EDAD ESTUDIOS HIJOS PAREJA SITUACION;

MISSING ARE ALL (99);

USEVARIABLES ARE PDISC1 PDISC2 PDISC3 PDISC5;

CATEGORICAL ARE PDISC1 PDISC2 PDISC3 PDISC5;

ANALYSIS:

ESTIMATOR IS WLSMV;

ITERATIONS = 1000;

CONVERGENCE = 0.005;

COVERAGE = 0.10;

MODEL:

PDISC BY PDISC1 PDISC2 PDISC3 PDISC5;

OUTPUT:

SAMPSTAT RESIDUAL STANDARDIZED MODINDICES (ALL);

**MM_PDISCmg.inp**

DATA:

FILE IS "datitems99.DAT";

FORMAT IS FREE;

TYPE IS INDIVIDUAL;

NGROUPS = 2;

NOBSERVATIONS ARE 853;

VARIABLE:

NAMES ARE cv1 cv2 cv3 cv4 cv5 cv6 cv7 cv8 cv9 cv10 cv11 cv12 cv13 cv14

cv15 cv16 cv17 cv18 cv19 cv20 cv21 cv22 cv23 cv24 cv25 cv26

sf1 sf2 sf3 sf4 sf5 sf6 sf7 sf8 sf9 sf10 sf11 sf12

GHQ121 GHQ122 GHQ123 GHQ124 GHQ125 GHQ126 GHQ127 GHQ128

GHQ129 GHQ1210 GHQ1211 GHQ1212

di1 di2 di3 di4 di5

keyes1 keyes2 keyes3 keyes4 keyes5 keyes6 keyes7 keyes8 keyes9 keyes10

keyes11 keyes12

keyes13 keyes14 keyes15 keyes16 keyes17 PWB1 PWB2 PWB3 PWB4 PWB5

PWB6 PWB7 PWB8

PWB9 PWB10 PWB11 PWB12 PWB13 PWB14 PWB15 PWB16 PWB17 PWB18

PWB19 PWB20 PWB21

PWB22 PWB23 PWB24 PWB25 PWB26 PWB27 PWB28 PWB29

ruiz1 ruiz2 ruiz3 ruiz4 ruiz5 ruiz6 ruiz7 ruiz8 ruiz9 ruiz10 ruiz11

ruiz12 ruiz13 ruiz14 ruiz15

ruiz16 ruiz17 ruiz18 ruiz19 ruiz20 ruiz21 ruiz22 ruiz23 ruiz24

oq1 oq2 oq3 oq4 oq5 oq6 oq7 oq8 oq9 oq10 oq11 oq12 oq13 oq14 oq15 oq16

oq17 oq18 oq19 oq20

oq21 oq22 oq23 oq24 oq25 oq26 oq27 oq28 oq29 oq30 oq31 oq32 oq33 oq34

oq35 oq36 oq37 oq38 oq39

oq40 oq41 oq42 oq43 oq44 oq45

DSE1 DSE2 DSE3 DSE4 DSE5 DSE6 DSE7 DSE8 DSE9 DSE10

V181 V182

V183 V184 V185 V186

AUTCOL1 AUTCOL2 AUTCOL3 AUTCOL4 AUTCOL5 AUTCOL6

AUTOSIM

V194A V194B V194C

V195A V195B V195C

V196A V196B V196C V196D

V197A V197B V197C V197D V197E V197F

V198A V198B V198C V198D V198E V198F V198G V198H V198I V198J V198K V198L

V199 V200

PDISC1 PDISC2 PDISC3 PDISC4 PDISC5

V206 V207

V208 V209A

V209B V209C V209D V209E V209F V209G V210 V211A1 V211A2 V211B1 V211B2 V211C1

V211C2 V211D1 V211D2

V211E1 V211E2 V211F1 V211F2 V211G1 V211G2 V212 V213 V214 V215 V216 V217 V218

V219 V220 V221 V222

V223 V224

V225 V226 V227 V228 V229 V230 V231 V232 V233 V234 V235 V236 V237 V238_1

V238_2 V239 V240 V241 V242

V243 V244 V245 V246 V247 V248 V249 V250 V251 V252 V253 V254 V255 V256 V257

V258 V259 V260

V261 V262 V263 V264 V265 V266 V267 V268 V269 V270 V271 V272 V273 V274 V275

V276 V277 V278 V279 V280

V281 V282 V283 V284 V285 V286 V287 V288 V289 V290 V291 V292 V293A

V294 V295 V296 V297 V298 V299 V300 V301 V302 V303 V304 V305 V306 V307 V308

V309 V310 V311

V312A V313 V314 V317

V318 V319 V320 V321 V322 V323 V324 V325 V326 V327

SEST1 SEST2 SEST3 SEST4 SEST5 SEST6 SEST7

V335 V336 V337 V338 V339 V340 V341 V342 V343 V344 V345 V346 V347 V348 V349 V350

V351 V352 V353 V354 V355 V356A V356B V356C V356D

V356E V356F V356G V356H V356I V356J V360A V360B V362A V363 V365 V367 V369A

V369B V370B FENOTIPO

CIUDAD PAIS SEXO EDAD ESTUDIOS HIJOS PAREJA SITUACION;

MISSING ARE ALL (99);

USEVARIABLES ARE PDISC1 PDISC2 PDISC3 PDISC5;

GROUPING IS PAIS (1=COL 2=PER);

CATEGORICAL ARE PDISC1 PDISC2 PDISC3 PDISC5;

ANALYSIS:

ESTIMATOR IS WLSMV;

ITERATIONS = 1000;

CONVERGENCE = 0.005;

COVERAGE = 0.10;

MODEL:

PDISC BY PDISC1 PDISC2 PDISC3 PDISC5;

OUTPUT:

SAMPSTAT RESIDUAL STANDARDIZED MODINDICES (ALL);

**MM_PWB.inp**

DATA:

FILE IS "datitems99.DAT";

FORMAT IS FREE;

TYPE IS INDIVIDUAL;

NOBSERVATIONS ARE 853;

VARIABLE:

NAMES ARE cv1 cv2 cv3 cv4 cv5 cv6 cv7 cv8 cv9 cv10 cv11 cv12 cv13 cv14

cv15 cv16 cv17 cv18 cv19 cv20 cv21 cv22 cv23 cv24 cv25 cv26

sf1 sf2 sf3 sf4 sf5 sf6 sf7 sf8 sf9 sf10 sf11 sf12

GHQ121 GHQ122 GHQ123 GHQ124 GHQ125 GHQ126 GHQ127 GHQ128

GHQ129 GHQ1210 GHQ1211 GHQ1212

di1 di2 di3 di4 di5

keyes1 keyes2 keyes3 keyes4 keyes5 keyes6 keyes7 keyes8 keyes9 keyes10

keyes11 keyes12

keyes13 keyes14 keyes15 keyes16 keyes17 PWB1 PWB2 PWB3 PWB4 PWB5

PWB6 PWB7 PWB8

PWB9 PWB10 PWB11 PWB12 PWB13 PWB14 PWB15 PWB16 PWB17 PWB18

PWB19 PWB20 PWB21

PWB22 PWB23 PWB24 PWB25 PWB26 PWB27 PWB28 PWB29

ruiz1 ruiz2 ruiz3 ruiz4 ruiz5 ruiz6 ruiz7 ruiz8 ruiz9 ruiz10 ruiz11

ruiz12 ruiz13 ruiz14 ruiz15

ruiz16 ruiz17 ruiz18 ruiz19 ruiz20 ruiz21 ruiz22 ruiz23 ruiz24

oq1 oq2 oq3 oq4 oq5 oq6 oq7 oq8 oq9 oq10 oq11 oq12 oq13 oq14 oq15 oq16

oq17 oq18 oq19 oq20

oq21 oq22 oq23 oq24 oq25 oq26 oq27 oq28 oq29 oq30 oq31 oq32 oq33 oq34

oq35 oq36 oq37 oq38 oq39

oq40 oq41 oq42 oq43 oq44 oq45

DSE1 DSE2 DSE3 DSE4 DSE5 DSE6 DSE7 DSE8 DSE9 DSE10

V181 V182

V183 V184 V185 V186

AUTCOL1 AUTCOL2 AUTCOL3 AUTCOL4 AUTCOL5 AUTCOL6

AUTOSIM

V194A V194B V194C

V195A V195B V195C

V196A V196B V196C V196D

V197A V197B V197C V197D V197E V197F

V198A V198B V198C V198D V198E V198F V198G V198H V198I V198J V198K V198L

V199 V200

PDISC1 PDISC2 PDISC3 PDISC4 PDISC5

V206 V207

V208 V209A

V209B V209C V209D V209E V209F V209G V210 V211A1 V211A2 V211B1 V211B2 V211C1

V211C2 V211D1 V211D2

V211E1 V211E2 V211F1 V211F2 V211G1 V211G2 V212 V213 V214 V215 V216 V217 V218

V219 V220 V221 V222

V223 V224

V225 V226 V227 V228 V229 V230 V231 V232 V233 V234 V235 V236 V237 V238_1

V238_2 V239 V240 V241 V242

V243 V244 V245 V246 V247 V248 V249 V250 V251 V252 V253 V254 V255 V256 V257

V258 V259 V260

V261 V262 V263 V264 V265 V266 V267 V268 V269 V270 V271 V272 V273 V274 V275

V276 V277 V278 V279 V280

V281 V282 V283 V284 V285 V286 V287 V288 V289 V290 V291 V292 V293A

V294 V295 V296 V297 V298 V299 V300 V301 V302 V303 V304 V305 V306 V307 V308

V309 V310 V311

V312A V313 V314 V317

V318 V319 V320 V321 V322 V323 V324 V325 V326 V327

SEST1 SEST2 SEST3 SEST4 SEST5 SEST6 SEST7

V335 V336 V337 V338 V339 V340 V341 V342 V343 V344 V345 V346 V347 V348 V349 V350

V351 V352 V353 V354 V355 V356A V356B V356C V356D

V356E V356F V356G V356H V356I V356J V360A V360B V362A V363 V365 V367 V369A

V369B V370B FENOTIPO

CIUDAD PAIS SEXO EDAD ESTUDIOS HIJOS PAREJA SITUACION;

MISSING ARE ALL (99);

USEVARIABLES ARE PWB1 PWB4 PWB6

PWB7 PWB8 PWB10 PWB11 PWB12 PWB14

PWB15 PWB16 PWB17 PWB18 PWB20 PWB21

PWB22 PWB24 PWB25 PWB27 PWB28 PWB29;

CATEGORICAL ARE PWB1 PWB4 PWB6

PWB7 PWB8 PWB10 PWB11 PWB12 PWB14

PWB15 PWB16 PWB17 PWB18 PWB20 PWB21

PWB22 PWB24 PWB25 PWB27 PWB28 PWB29;

ANALYSIS:

ESTIMATOR IS WLSMV;

ITERATIONS = 1000;

CONVERGENCE = 0.00005;

COVERAGE = 0.10;

MODEL:

PWB_SA BY PWB1 PWB7 PWB17 PWB24;

PWB_PR BY PWB8 PWB12 PWB22 PWB25;

PWB_AU BY PWB4 PWB18;

PWB_DE BY PWB10 PWB14 PWB29;

PWB_PG BY PWB21 PWB27 PWB28;

PWB_PO BY PWB6 PWB11 PWB15 PWB16 PWB20;

OUTPUT:

SAMPSTAT RESIDUAL STANDARDIZED MODINDICES;

**MM_PWBmg.inp**

DATA:

FILE IS "datitems99.DAT";

FORMAT IS FREE;

TYPE IS INDIVIDUAL;

NGROUPS = 2;

NOBSERVATIONS ARE 853;

VARIABLE:

NAMES ARE cv1 cv2 cv3 cv4 cv5 cv6 cv7 cv8 cv9 cv10 cv11 cv12 cv13 cv14

cv15 cv16 cv17 cv18 cv19 cv20 cv21 cv22 cv23 cv24 cv25 cv26

sf1 sf2 sf3 sf4 sf5 sf6 sf7 sf8 sf9 sf10 sf11 sf12

GHQ121 GHQ122 GHQ123 GHQ124 GHQ125 GHQ126 GHQ127 GHQ128

GHQ129 GHQ1210 GHQ1211 GHQ1212

di1 di2 di3 di4 di5

keyes1 keyes2 keyes3 keyes4 keyes5 keyes6 keyes7 keyes8 keyes9 keyes10

keyes11 keyes12

keyes13 keyes14 keyes15 keyes16 keyes17 PWB1 PWB2 PWB3 PWB4 PWB5

PWB6 PWB7 PWB8

PWB9 PWB10 PWB11 PWB12 PWB13 PWB14 PWB15 PWB16 PWB17 PWB18

PWB19 PWB20 PWB21

PWB22 PWB23 PWB24 PWB25 PWB26 PWB27 PWB28 PWB29

ruiz1 ruiz2 ruiz3 ruiz4 ruiz5 ruiz6 ruiz7 ruiz8 ruiz9 ruiz10 ruiz11

ruiz12 ruiz13 ruiz14 ruiz15

ruiz16 ruiz17 ruiz18 ruiz19 ruiz20 ruiz21 ruiz22 ruiz23 ruiz24

oq1 oq2 oq3 oq4 oq5 oq6 oq7 oq8 oq9 oq10 oq11 oq12 oq13 oq14 oq15 oq16

oq17 oq18 oq19 oq20

oq21 oq22 oq23 oq24 oq25 oq26 oq27 oq28 oq29 oq30 oq31 oq32 oq33 oq34

oq35 oq36 oq37 oq38 oq39

oq40 oq41 oq42 oq43 oq44 oq45

DSE1 DSE2 DSE3 DSE4 DSE5 DSE6 DSE7 DSE8 DSE9 DSE10

V181 V182

V183 V184 V185 V186

AUTCOL1 AUTCOL2 AUTCOL3 AUTCOL4 AUTCOL5 AUTCOL6

AUTOSIM

V194A V194B V194C

V195A V195B V195C

V196A V196B V196C V196D

V197A V197B V197C V197D V197E V197F

V198A V198B V198C V198D V198E V198F V198G V198H V198I V198J V198K V198L

V199 V200

PDISC1 PDISC2 PDISC3 PDISC4 PDISC5

V206 V207

V208 V209A

V209B V209C V209D V209E V209F V209G V210 V211A1 V211A2 V211B1 V211B2 V211C1

V211C2 V211D1 V211D2

V211E1 V211E2 V211F1 V211F2 V211G1 V211G2 V212 V213 V214 V215 V216 V217 V218

V219 V220 V221 V222

V223 V224

V225 V226 V227 V228 V229 V230 V231 V232 V233 V234 V235 V236 V237 V238_1

V238_2 V239 V240 V241 V242

V243 V244 V245 V246 V247 V248 V249 V250 V251 V252 V253 V254 V255 V256 V257

V258 V259 V260

V261 V262 V263 V264 V265 V266 V267 V268 V269 V270 V271 V272 V273 V274 V275

V276 V277 V278 V279 V280

V281 V282 V283 V284 V285 V286 V287 V288 V289 V290 V291 V292 V293A

V294 V295 V296 V297 V298 V299 V300 V301 V302 V303 V304 V305 V306 V307 V308

V309 V310 V311

V312A V313 V314 V317

V318 V319 V320 V321 V322 V323 V324 V325 V326 V327

SEST1 SEST2 SEST3 SEST4 SEST5 SEST6 SEST7

V335 V336 V337 V338 V339 V340 V341 V342 V343 V344 V345 V346 V347 V348 V349 V350

V351 V352 V353 V354 V355 V356A V356B V356C V356D

V356E V356F V356G V356H V356I V356J V360A V360B V362A V363 V365 V367 V369A

V369B V370B FENOTIPO

CIUDAD PAIS SEXO EDAD ESTUDIOS HIJOS PAREJA SITUACION;

MISSING ARE ALL (99);

USEVARIABLES ARE PWB1 PWB4 PWB6

PWB7 PWB8 PWB10 PWB11 PWB12 PWB14

PWB15 PWB16 PWB17 PWB18 PWB20 PWB21

PWB22 PWB24 PWB25 PWB27 PWB28 PWB29;

GROUPING IS PAIS (1=COL 2=PER);

CATEGORICAL ARE PWB1 PWB4 PWB6

PWB7 PWB8 PWB10 PWB11 PWB12 PWB14

PWB15 PWB16 PWB17 PWB18 PWB20 PWB21

PWB22 PWB24 PWB25 PWB27 PWB28 PWB29;

ANALYSIS:

ESTIMATOR IS WLSMV;

ITERATIONS = 1000;

CONVERGENCE = 0.005;

COVERAGE = 0.10;

Model= configural metric scalar;

MODEL:

PWB_SA BY PWB1 PWB7 PWB17 PWB24;

PWB_PR BY PWB8 PWB12 PWB22 PWB25;

PWB_AU BY PWB4 PWB18;

PWB_DE BY PWB10 PWB14 PWB29;

PWB_PG BY PWB21 PWB27 PWB28;

PWB_PO BY PWB6 PWB11 PWB15 PWB16 PWB20;

OUTPUT:

SAMPSTAT RESIDUAL STANDARDIZED MODINDICES;

**MM_SEST.inp**

DATA:

FILE IS "datitems99.DAT";

FORMAT IS FREE;

TYPE IS INDIVIDUAL;

NOBSERVATIONS ARE 853;

VARIABLE:

NAMES ARE cv1 cv2 cv3 cv4 cv5 cv6 cv7 cv8 cv9 cv10 cv11 cv12 cv13 cv14

cv15 cv16 cv17 cv18 cv19 cv20 cv21 cv22 cv23 cv24 cv25 cv26

sf1 sf2 sf3 sf4 sf5 sf6 sf7 sf8 sf9 sf10 sf11 sf12

GHQ121 GHQ122 GHQ123 GHQ124 GHQ125 GHQ126 GHQ127 GHQ128

GHQ129 GHQ1210 GHQ1211 GHQ1212

di1 di2 di3 di4 di5

keyes1 keyes2 keyes3 keyes4 keyes5 keyes6 keyes7 keyes8 keyes9 keyes10

keyes11 keyes12

keyes13 keyes14 keyes15 keyes16 keyes17 PWB1 PWB2 PWB3 PWB4 PWB5

PWB6 PWB7 PWB8

PWB9 PWB10 PWB11 PWB12 PWB13 PWB14 PWB15 PWB16 PWB17 PWB18

PWB19 PWB20 PWB21

PWB22 PWB23 PWB24 PWB25 PWB26 PWB27 PWB28 PWB29

ruiz1 ruiz2 ruiz3 ruiz4 ruiz5 ruiz6 ruiz7 ruiz8 ruiz9 ruiz10 ruiz11

ruiz12 ruiz13 ruiz14 ruiz15

ruiz16 ruiz17 ruiz18 ruiz19 ruiz20 ruiz21 ruiz22 ruiz23 ruiz24

oq1 oq2 oq3 oq4 oq5 oq6 oq7 oq8 oq9 oq10 oq11 oq12 oq13 oq14 oq15 oq16

oq17 oq18 oq19 oq20

oq21 oq22 oq23 oq24 oq25 oq26 oq27 oq28 oq29 oq30 oq31 oq32 oq33 oq34

oq35 oq36 oq37 oq38 oq39

oq40 oq41 oq42 oq43 oq44 oq45

DSE1 DSE2 DSE3 DSE4 DSE5 DSE6 DSE7 DSE8 DSE9 DSE10

V181 V182

V183 V184 V185 V186

AUTCOL1 AUTCOL2 AUTCOL3 AUTCOL4 AUTCOL5 AUTCOL6

AUTOSIM

V194A V194B V194C

V195A V195B V195C

V196A V196B V196C V196D

V197A V197B V197C V197D V197E V197F

V198A V198B V198C V198D V198E V198F V198G V198H V198I V198J V198K V198L

V199 V200

PDISC1 PDISC2 PDISC3 PDISC4 PDISC5

V206 V207

V208 V209A

V209B V209C V209D V209E V209F V209G V210 V211A1 V211A2 V211B1 V211B2 V211C1

V211C2 V211D1 V211D2

V211E1 V211E2 V211F1 V211F2 V211G1 V211G2 V212 V213 V214 V215 V216 V217 V218

V219 V220 V221 V222

V223 V224

V225 V226 V227 V228 V229 V230 V231 V232 V233 V234 V235 V236 V237 V238_1

V238_2 V239 V240 V241 V242

V243 V244 V245 V246 V247 V248 V249 V250 V251 V252 V253 V254 V255 V256 V257

V258 V259 V260

V261 V262 V263 V264 V265 V266 V267 V268 V269 V270 V271 V272 V273 V274 V275

V276 V277 V278 V279 V280

V281 V282 V283 V284 V285 V286 V287 V288 V289 V290 V291 V292 V293A

V294 V295 V296 V297 V298 V299 V300 V301 V302 V303 V304 V305 V306 V307 V308

V309 V310 V311

V312A V313 V314 V317

V318 V319 V320 V321 V322 V323 V324 V325 V326 V327

SEST1 SEST2 SEST3 SEST4 SEST5 SEST6 SEST7

V335 V336 V337 V338 V339 V340 V341 V342 V343 V344 V345 V346 V347 V348 V349 V350

V351 V352 V353 V354 V355 V356A V356B V356C V356D

V356E V356F V356G V356H V356I V356J V360A V360B V362A V363 V365 V367 V369A

V369B V370B FENOTIPO

CIUDAD PAIS SEXO EDAD ESTUDIOS HIJOS PAREJA SITUACION;

MISSING ARE ALL (99);

USEVARIABLES ARE SEST1 SEST2 SEST3 SEST4 SEST5 SEST6 SEST7;

CATEGORICAL ARE SEST1 SEST2 SEST3 SEST4 SEST5 SEST6 SEST7;

ANALYSIS:

ESTIMATOR IS WLSMV;

ITERATIONS = 1000;

CONVERGENCE = 0.005;

COVERAGE = 0.10;

MODEL:

SEST BY SEST1 SEST2 SEST3 SEST4 SEST5 SEST6 SEST7;

OUTPUT:

SAMPSTAT RESIDUAL STANDARDIZED MODINDICES (ALL);

**MM_SESTmg.inp**

DATA:

FILE IS "datitems99.DAT";

FORMAT IS FREE;

TYPE IS INDIVIDUAL;

NGROUPS = 2;

NOBSERVATIONS ARE 853;

VARIABLE:

NAMES ARE cv1 cv2 cv3 cv4 cv5 cv6 cv7 cv8 cv9 cv10 cv11 cv12 cv13 cv14

cv15 cv16 cv17 cv18 cv19 cv20 cv21 cv22 cv23 cv24 cv25 cv26

sf1 sf2 sf3 sf4 sf5 sf6 sf7 sf8 sf9 sf10 sf11 sf12

GHQ121 GHQ122 GHQ123 GHQ124 GHQ125 GHQ126 GHQ127 GHQ128

GHQ129 GHQ1210 GHQ1211 GHQ1212

di1 di2 di3 di4 di5

keyes1 keyes2 keyes3 keyes4 keyes5 keyes6 keyes7 keyes8 keyes9 keyes10

keyes11 keyes12

keyes13 keyes14 keyes15 keyes16 keyes17 PWB1 PWB2 PWB3 PWB4 PWB5

PWB6 PWB7 PWB8

PWB9 PWB10 PWB11 PWB12 PWB13 PWB14 PWB15 PWB16 PWB17 PWB18

PWB19 PWB20 PWB21

PWB22 PWB23 PWB24 PWB25 PWB26 PWB27 PWB28 PWB29

ruiz1 ruiz2 ruiz3 ruiz4 ruiz5 ruiz6 ruiz7 ruiz8 ruiz9 ruiz10 ruiz11

ruiz12 ruiz13 ruiz14 ruiz15

ruiz16 ruiz17 ruiz18 ruiz19 ruiz20 ruiz21 ruiz22 ruiz23 ruiz24

oq1 oq2 oq3 oq4 oq5 oq6 oq7 oq8 oq9 oq10 oq11 oq12 oq13 oq14 oq15 oq16

oq17 oq18 oq19 oq20

oq21 oq22 oq23 oq24 oq25 oq26 oq27 oq28 oq29 oq30 oq31 oq32 oq33 oq34

oq35 oq36 oq37 oq38 oq39

oq40 oq41 oq42 oq43 oq44 oq45

DSE1 DSE2 DSE3 DSE4 DSE5 DSE6 DSE7 DSE8 DSE9 DSE10

V181 V182

V183 V184 V185 V186

AUTCOL1 AUTCOL2 AUTCOL3 AUTCOL4 AUTCOL5 AUTCOL6

AUTOSIM

V194A V194B V194C

V195A V195B V195C

V196A V196B V196C V196D

V197A V197B V197C V197D V197E V197F

V198A V198B V198C V198D V198E V198F V198G V198H V198I V198J V198K V198L

V199 V200

PDISC1 PDISC2 PDISC3 PDISC4 PDISC5

V206 V207

V208 V209A

V209B V209C V209D V209E V209F V209G V210 V211A1 V211A2 V211B1 V211B2 V211C1

V211C2 V211D1 V211D2

V211E1 V211E2 V211F1 V211F2 V211G1 V211G2 V212 V213 V214 V215 V216 V217 V218

V219 V220 V221 V222

V223 V224

V225 V226 V227 V228 V229 V230 V231 V232 V233 V234 V235 V236 V237 V238_1

V238_2 V239 V240 V241 V242

V243 V244 V245 V246 V247 V248 V249 V250 V251 V252 V253 V254 V255 V256 V257

V258 V259 V260

V261 V262 V263 V264 V265 V266 V267 V268 V269 V270 V271 V272 V273 V274 V275

V276 V277 V278 V279 V280

V281 V282 V283 V284 V285 V286 V287 V288 V289 V290 V291 V292 V293A

V294 V295 V296 V297 V298 V299 V300 V301 V302 V303 V304 V305 V306 V307 V308

V309 V310 V311

V312A V313 V314 V317

V318 V319 V320 V321 V322 V323 V324 V325 V326 V327

SEST1 SEST2 SEST3 SEST4 SEST5 SEST6 SEST7

V335 V336 V337 V338 V339 V340 V341 V342 V343 V344 V345 V346 V347 V348 V349 V350

V351 V352 V353 V354 V355 V356A V356B V356C V356D

V356E V356F V356G V356H V356I V356J V360A V360B V362A V363 V365 V367 V369A

V369B V370B FENOTIPO

CIUDAD PAIS SEXO EDAD ESTUDIOS HIJOS PAREJA SITUACION;

MISSING ARE ALL (99);

USEVARIABLES ARE SEST1 SEST2 SEST3 SEST5;

GROUPING IS PAIS (1=COL 2=PER);

CATEGORICAL ARE SEST1 SEST2 SEST3 SEST5;

ANALYSIS:

ESTIMATOR IS WLSMV;

ITERATIONS = 1000;

CONVERGENCE = 0.005;

COVERAGE = 0.10;

Model= configural metric scalar;

MODEL:

SEST BY SEST1 SEST2 SEST3 SEST5;

OUTPUT:

SAMPSTAT RESIDUAL STANDARDIZED MODINDICES (ALL);

**SEM_DISCPWB.inp**

DATA:

FILE IS "datitems99.DAT";

FORMAT IS FREE;

TYPE IS INDIVIDUAL;

NOBSERVATIONS ARE 853;

VARIABLE:

NAMES ARE cv1 cv2 cv3 cv4 cv5 cv6 cv7 cv8 cv9 cv10 cv11 cv12 cv13 cv14

cv15 cv16 cv17 cv18 cv19 cv20 cv21 cv22 cv23 cv24 cv25 cv26

sf1 sf2 sf3 sf4 sf5 sf6 sf7 sf8 sf9 sf10 sf11 sf12

GHQ121 GHQ122 GHQ123 GHQ124 GHQ125 GHQ126 GHQ127 GHQ128

GHQ129 GHQ1210 GHQ1211 GHQ1212

di1 di2 di3 di4 di5

keyes1 keyes2 keyes3 keyes4 keyes5 keyes6 keyes7 keyes8 keyes9 keyes10

keyes11 keyes12

keyes13 keyes14 keyes15 keyes16 keyes17 PWB1 PWB2 PWB3 PWB4 PWB5

PWB6 PWB7 PWB8

PWB9 PWB10 PWB11 PWB12 PWB13 PWB14 PWB15 PWB16 PWB17 PWB18

PWB19 PWB20 PWB21

PWB22 PWB23 PWB24 PWB25 PWB26 PWB27 PWB28 PWB29

ruiz1 ruiz2 ruiz3 ruiz4 ruiz5 ruiz6 ruiz7 ruiz8 ruiz9 ruiz10 ruiz11

ruiz12 ruiz13 ruiz14 ruiz15

ruiz16 ruiz17 ruiz18 ruiz19 ruiz20 ruiz21 ruiz22 ruiz23 ruiz24

oq1 oq2 oq3 oq4 oq5 oq6 oq7 oq8 oq9 oq10 oq11 oq12 oq13 oq14 oq15 oq16

oq17 oq18 oq19 oq20

oq21 oq22 oq23 oq24 oq25 oq26 oq27 oq28 oq29 oq30 oq31 oq32 oq33 oq34

oq35 oq36 oq37 oq38 oq39

oq40 oq41 oq42 oq43 oq44 oq45

DSE1 DSE2 DSE3 DSE4 DSE5 DSE6 DSE7 DSE8 DSE9 DSE10

V181 V182

V183 V184 V185 V186

AUTCOL1 AUTCOL2 AUTCOL3 AUTCOL4 AUTCOL5 AUTCOL6

AUTOSIM

V194A V194B V194C

V195A V195B V195C

V196A V196B V196C V196D

V197A V197B V197C V197D V197E V197F

V198A V198B V198C V198D V198E V198F V198G V198H V198I V198J V198K V198L

V199 V200

PDISC1 PDISC2 PDISC3 PDISC4 PDISC5

V206 V207

V208 V209A

V209B V209C V209D V209E V209F V209G V210 V211A1 V211A2 V211B1 V211B2 V211C1

V211C2 V211D1 V211D2

V211E1 V211E2 V211F1 V211F2 V211G1 V211G2 V212 V213 V214 V215 V216 V217 V218

V219 V220 V221 V222

V223 V224

V225 V226 V227 V228 V229 V230 V231 V232 V233 V234 V235 V236 V237 V238_1

V238_2 V239 V240 V241 V242

V243 V244 V245 V246 V247 V248 V249 V250 V251 V252 V253 V254 V255 V256 V257

V258 V259 V260

V261 V262 V263 V264 V265 V266 V267 V268 V269 V270 V271 V272 V273 V274 V275

V276 V277 V278 V279 V280

V281 V282 V283 V284 V285 V286 V287 V288 V289 V290 V291 V292 V293A

V294 V295 V296 V297 V298 V299 V300 V301 V302 V303 V304 V305 V306 V307 V308

V309 V310 V311

V312A V313 V314 V317

V318 V319 V320 V321 V322 V323 V324 V325 V326 V327

SEST1 SEST2 SEST3 SEST4 SEST5 SEST6 SEST7

V335 V336 V337 V338 V339 V340 V341 V342 V343 V344 V345 V346 V347 V348 V349 V350

V351 V352 V353 V354 V355 V356A V356B V356C V356D

V356E V356F V356G V356H V356I V356J V360A V360B V362A V363 V365 V367 V369A

V369B V370B FENOTIPO

CIUDAD PAIS SEXO EDAD ESTUDIOS HIJOS PAREJA SITUACION;

MISSING ARE ALL (99);

USEVARIABLES ARE PWB1 PWB4 PWB6 PWB7 PWB8

PWB10 PWB11 PWB12 PWB14 PWB15 PWB16 PWB17 PWB18

PWB20 PWB21

PWB22 PWB24 PWB25 PWB27 PWB28 PWB29

PDISC1 PDISC2 PDISC3 PDISC5;

CATEGORICAL ARE PWB1 PWB4 PWB6 PWB7 PWB8

PWB10 PWB11 PWB12 PWB14 PWB15 PWB16 PWB17 PWB18

PWB20 PWB21

PWB22 PWB24 PWB25 PWB27 PWB28 PWB29

PDISC1 PDISC2 PDISC3 PDISC5;

ANALYSIS:

ESTIMATOR IS WLSMV;

ITERATIONS = 1000;

CONVERGENCE = 0.005;

COVERAGE = 0.10;

MODEL:

PDISC BY PDISC1 PDISC2 PDISC3 PDISC5;

PWB_SA BY PWB1 PWB7 PWB17 PWB24;

PWB_PR BY PWB8 PWB12 PWB22 PWB25;

PWB_AU BY PWB4 PWB18;

PWB_DE BY PWB10 PWB14 PWB29;

PWB_PG BY PWB21 PWB27 PWB28;

PWB_PO BY PWB6 PWB11 PWB15 PWB16 PWB20;

PWB_SA ON PDISC;

PWB_PR ON PDISC;

PWB_AU ON PDISC;

PWB_DE ON PDISC;

PWB_PG ON PDISC;

PWB_PO ON PDISC;

OUTPUT:

SAMPSTAT RESIDUAL STANDARDIZED(STDYX);

**SEM_DISCSEST.inp**

DATA:

FILE IS "datitems99.DAT";

FORMAT IS FREE;

TYPE IS INDIVIDUAL;

NOBSERVATIONS ARE 853;

VARIABLE:

NAMES ARE cv1 cv2 cv3 cv4 cv5 cv6 cv7 cv8 cv9 cv10 cv11 cv12 cv13 cv14

cv15 cv16 cv17 cv18 cv19 cv20 cv21 cv22 cv23 cv24 cv25 cv26

sf1 sf2 sf3 sf4 sf5 sf6 sf7 sf8 sf9 sf10 sf11 sf12

GHQ121 GHQ122 GHQ123 GHQ124 GHQ125 GHQ126 GHQ127 GHQ128

GHQ129 GHQ1210 GHQ1211 GHQ1212

di1 di2 di3 di4 di5

keyes1 keyes2 keyes3 keyes4 keyes5 keyes6 keyes7 keyes8 keyes9 keyes10

keyes11 keyes12

keyes13 keyes14 keyes15 keyes16 keyes17 PWB1 PWB2 PWB3 PWB4 PWB5

PWB6 PWB7 PWB8

PWB9 PWB10 PWB11 PWB12 PWB13 PWB14 PWB15 PWB16 PWB17 PWB18

PWB19 PWB20 PWB21

PWB22 PWB23 PWB24 PWB25 PWB26 PWB27 PWB28 PWB29

ruiz1 ruiz2 ruiz3 ruiz4 ruiz5 ruiz6 ruiz7 ruiz8 ruiz9 ruiz10 ruiz11

ruiz12 ruiz13 ruiz14 ruiz15

ruiz16 ruiz17 ruiz18 ruiz19 ruiz20 ruiz21 ruiz22 ruiz23 ruiz24

oq1 oq2 oq3 oq4 oq5 oq6 oq7 oq8 oq9 oq10 oq11 oq12 oq13 oq14 oq15 oq16

oq17 oq18 oq19 oq20

oq21 oq22 oq23 oq24 oq25 oq26 oq27 oq28 oq29 oq30 oq31 oq32 oq33 oq34

oq35 oq36 oq37 oq38 oq39

oq40 oq41 oq42 oq43 oq44 oq45

DSE1 DSE2 DSE3 DSE4 DSE5 DSE6 DSE7 DSE8 DSE9 DSE10

V181 V182

V183 V184 V185 V186

AUTCOL1 AUTCOL2 AUTCOL3 AUTCOL4 AUTCOL5 AUTCOL6

AUTOSIM

V194A V194B V194C

V195A V195B V195C

V196A V196B V196C V196D

V197A V197B V197C V197D V197E V197F

V198A V198B V198C V198D V198E V198F V198G V198H V198I V198J V198K V198L

V199 V200

PDISC1 PDISC2 PDISC3 PDISC4 PDISC5

V206 V207

V208 V209A

V209B V209C V209D V209E V209F V209G V210 V211A1 V211A2 V211B1 V211B2 V211C1

V211C2 V211D1 V211D2

V211E1 V211E2 V211F1 V211F2 V211G1 V211G2 V212 V213 V214 V215 V216 V217 V218

V219 V220 V221 V222

V223 V224

V225 V226 V227 V228 V229 V230 V231 V232 V233 V234 V235 V236 V237 V238_1

V238_2 V239 V240 V241 V242

V243 V244 V245 V246 V247 V248 V249 V250 V251 V252 V253 V254 V255 V256 V257

V258 V259 V260

V261 V262 V263 V264 V265 V266 V267 V268 V269 V270 V271 V272 V273 V274 V275

V276 V277 V278 V279 V280

V281 V282 V283 V284 V285 V286 V287 V288 V289 V290 V291 V292 V293A

V294 V295 V296 V297 V298 V299 V300 V301 V302 V303 V304 V305 V306 V307 V308

V309 V310 V311

V312A V313 V314 V317

V318 V319 V320 V321 V322 V323 V324 V325 V326 V327

SEST1 SEST2 SEST3 SEST4 SEST5 SEST6 SEST7

V335 V336 V337 V338 V339 V340 V341 V342 V343 V344 V345 V346 V347 V348 V349 V350

V351 V352 V353 V354 V355 V356A V356B V356C V356D

V356E V356F V356G V356H V356I V356J V360A V360B V362A V363 V365 V367 V369A

V369B V370B FENOTIPO

CIUDAD PAIS SEXO EDAD ESTUDIOS HIJOS PAREJA SITUACION;

MISSING ARE ALL (99);

USEVARIABLES ARE PDISC1 PDISC2 PDISC3 PDISC5

SEST1 SEST2 SEST3 SEST5;

CATEGORICAL ARE PDISC1 PDISC2 PDISC3 PDISC5

SEST1 SEST2 SEST3 SEST5;

ANALYSIS:

ESTIMATOR IS WLSMV;

ITERATIONS = 1000;

CONVERGENCE = 0.005;

COVERAGE = 0.10;

MODEL:

PDISC BY PDISC1 PDISC2 PDISC3 PDISC5;

SEST BY SEST1 SEST2 SEST3 SEST5;

SEST ON PDISC;

OUTPUT:

SAMPSTAT RESIDUAL STANDARDIZED(STDYX);

**SEM_DISCSESTPWB.inp**

DATA:

FILE IS "datitems99.DAT";

FORMAT IS FREE;

TYPE IS INDIVIDUAL;

NOBSERVATIONS ARE 853;

VARIABLE:

NAMES ARE cv1 cv2 cv3 cv4 cv5 cv6 cv7 cv8 cv9 cv10 cv11 cv12 cv13 cv14

cv15 cv16 cv17 cv18 cv19 cv20 cv21 cv22 cv23 cv24 cv25 cv26

sf1 sf2 sf3 sf4 sf5 sf6 sf7 sf8 sf9 sf10 sf11 sf12

GHQ121 GHQ122 GHQ123 GHQ124 GHQ125 GHQ126 GHQ127 GHQ128

GHQ129 GHQ1210 GHQ1211 GHQ1212

di1 di2 di3 di4 di5

keyes1 keyes2 keyes3 keyes4 keyes5 keyes6 keyes7 keyes8 keyes9 keyes10

keyes11 keyes12

keyes13 keyes14 keyes15 keyes16 keyes17 PWB1 PWB2 PWB3 PWB4 PWB5

PWB6 PWB7 PWB8

PWB9 PWB10 PWB11 PWB12 PWB13 PWB14 PWB15 PWB16 PWB17 PWB18

PWB19 PWB20 PWB21

PWB22 PWB23 PWB24 PWB25 PWB26 PWB27 PWB28 PWB29

ruiz1 ruiz2 ruiz3 ruiz4 ruiz5 ruiz6 ruiz7 ruiz8 ruiz9 ruiz10 ruiz11

ruiz12 ruiz13 ruiz14 ruiz15

ruiz16 ruiz17 ruiz18 ruiz19 ruiz20 ruiz21 ruiz22 ruiz23 ruiz24

oq1 oq2 oq3 oq4 oq5 oq6 oq7 oq8 oq9 oq10 oq11 oq12 oq13 oq14 oq15 oq16

oq17 oq18 oq19 oq20

oq21 oq22 oq23 oq24 oq25 oq26 oq27 oq28 oq29 oq30 oq31 oq32 oq33 oq34

oq35 oq36 oq37 oq38 oq39

oq40 oq41 oq42 oq43 oq44 oq45

DSE1 DSE2 DSE3 DSE4 DSE5 DSE6 DSE7 DSE8 DSE9 DSE10

V181 V182

V183 V184 V185 V186

AUTCOL1 AUTCOL2 AUTCOL3 AUTCOL4 AUTCOL5 AUTCOL6

AUTOSIM

V194A V194B V194C

V195A V195B V195C

V196A V196B V196C V196D

V197A V197B V197C V197D V197E V197F

V198A V198B V198C V198D V198E V198F V198G V198H V198I V198J V198K V198L

V199 V200

PDISC1 PDISC2 PDISC3 PDISC4 PDISC5

V206 V207

V208 V209A

V209B V209C V209D V209E V209F V209G V210 V211A1 V211A2 V211B1 V211B2 V211C1

V211C2 V211D1 V211D2

V211E1 V211E2 V211F1 V211F2 V211G1 V211G2 V212 V213 V214 V215 V216 V217 V218

V219 V220 V221 V222

V223 V224

V225 V226 V227 V228 V229 V230 V231 V232 V233 V234 V235 V236 V237 V238_1

V238_2 V239 V240 V241 V242

V243 V244 V245 V246 V247 V248 V249 V250 V251 V252 V253 V254 V255 V256 V257

V258 V259 V260

V261 V262 V263 V264 V265 V266 V267 V268 V269 V270 V271 V272 V273 V274 V275

V276 V277 V278 V279 V280

V281 V282 V283 V284 V285 V286 V287 V288 V289 V290 V291 V292 V293A

V294 V295 V296 V297 V298 V299 V300 V301 V302 V303 V304 V305 V306 V307 V308

V309 V310 V311

V312A V313 V314 V317

V318 V319 V320 V321 V322 V323 V324 V325 V326 V327

SEST1 SEST2 SEST3 SEST4 SEST5 SEST6 SEST7

V335 V336 V337 V338 V339 V340 V341 V342 V343 V344 V345 V346 V347 V348 V349 V350

V351 V352 V353 V354 V355 V356A V356B V356C V356D

V356E V356F V356G V356H V356I V356J V360A V360B V362A V363 V365 V367 V369A

V369B V370B FENOTIPO

CIUDAD PAIS SEXO EDAD ESTUDIOS HIJOS PAREJA SITUACION;

MISSING ARE ALL (99);

USEVARIABLES ARE PWB1 PWB7 PWB17 PWB24

PWB8 PWB12 PWB22 PWB25

PWB4 PWB18 PWB10 PWB14 PWB29 PWB21 PWB27 PWB28

PWB6 PWB11 PWB15 PWB16

PWB20

PDISC1 PDISC2 PDISC3 PDISC5

SEST1 SEST2 SEST3 SEST5;

CATEGORICAL ARE PWB1 PWB7 PWB17 PWB24

PWB8 PWB12 PWB22 PWB25

PWB4 PWB18 PWB10 PWB14 PWB29 PWB21 PWB27 PWB28

PWB6 PWB11 PWB15 PWB16

PWB20

PDISC1 PDISC2 PDISC3 PDISC5

SEST1 SEST2 SEST3 SEST5;

ANALYSIS:

ESTIMATOR IS WLSMV;

ITERATIONS = 1000;

CONVERGENCE = 0.005;

COVERAGE = 0.10;

MODEL:

PDISC BY PDISC1 PDISC2 PDISC3 PDISC5;

SEST BY SEST1 SEST2 SEST3 SEST5;

PWB_SA BY PWB1 PWB7 PWB17 PWB24;

PWB_PR BY PWB8 PWB12 PWB22 PWB25;

PWB_AU BY PWB4 PWB18;

PWB_DE BY PWB10 PWB14 PWB29;

PWB_PG BY PWB21 PWB27 PWB28;

PWB_PO BY PWB6 PWB11 PWB15 PWB16 PWB20;

SEST ON PDISC;

PWB_SA ON PDISC;

PWB_PR ON PDISC;

PWB_AU ON PDISC;

PWB_DE ON PDISC;

PWB_PG ON PDISC;

PWB_PO ON PDISC;

PWB_SA ON SEST;

PWB_PR ON SEST;

PWB_AU ON SEST;

PWB_DE ON SEST;

PWB_PG ON SEST;

PWB_PO ON SEST;

OUTPUT:

SAMPSTAT RESIDUAL STANDARDIZED(STDYX);

**SEM_DISCSESTPWBmed.inp**

DATA:

FILE IS "datitems99.DAT";

FORMAT IS FREE;

TYPE IS INDIVIDUAL;

NOBSERVATIONS ARE 853;

VARIABLE:

NAMES ARE cv1 cv2 cv3 cv4 cv5 cv6 cv7 cv8 cv9 cv10 cv11 cv12 cv13 cv14

cv15 cv16 cv17 cv18 cv19 cv20 cv21 cv22 cv23 cv24 cv25 cv26

sf1 sf2 sf3 sf4 sf5 sf6 sf7 sf8 sf9 sf10 sf11 sf12

GHQ121 GHQ122 GHQ123 GHQ124 GHQ125 GHQ126 GHQ127 GHQ128

GHQ129 GHQ1210 GHQ1211 GHQ1212

di1 di2 di3 di4 di5

keyes1 keyes2 keyes3 keyes4 keyes5 keyes6 keyes7 keyes8 keyes9 keyes10

keyes11 keyes12

keyes13 keyes14 keyes15 keyes16 keyes17 PWB1 PWB2 PWB3 PWB4 PWB5

PWB6 PWB7 PWB8

PWB9 PWB10 PWB11 PWB12 PWB13 PWB14 PWB15 PWB16 PWB17 PWB18

PWB19 PWB20 PWB21

PWB22 PWB23 PWB24 PWB25 PWB26 PWB27 PWB28 PWB29

ruiz1 ruiz2 ruiz3 ruiz4 ruiz5 ruiz6 ruiz7 ruiz8 ruiz9 ruiz10 ruiz11

ruiz12 ruiz13 ruiz14 ruiz15

ruiz16 ruiz17 ruiz18 ruiz19 ruiz20 ruiz21 ruiz22 ruiz23 ruiz24

oq1 oq2 oq3 oq4 oq5 oq6 oq7 oq8 oq9 oq10 oq11 oq12 oq13 oq14 oq15 oq16

oq17 oq18 oq19 oq20

oq21 oq22 oq23 oq24 oq25 oq26 oq27 oq28 oq29 oq30 oq31 oq32 oq33 oq34

oq35 oq36 oq37 oq38 oq39

oq40 oq41 oq42 oq43 oq44 oq45

DSE1 DSE2 DSE3 DSE4 DSE5 DSE6 DSE7 DSE8 DSE9 DSE10

V181 V182

V183 V184 V185 V186

AUTCOL1 AUTCOL2 AUTCOL3 AUTCOL4 AUTCOL5 AUTCOL6

AUTOSIM

V194A V194B V194C

V195A V195B V195C

V196A V196B V196C V196D

V197A V197B V197C V197D V197E V197F

V198A V198B V198C V198D V198E V198F V198G V198H V198I V198J V198K V198L

V199 V200

PDISC1 PDISC2 PDISC3 PDISC4 PDISC5

V206 V207

V208 V209A

V209B V209C V209D V209E V209F V209G V210 V211A1 V211A2 V211B1 V211B2 V211C1

V211C2 V211D1 V211D2

V211E1 V211E2 V211F1 V211F2 V211G1 V211G2 V212 V213 V214 V215 V216 V217 V218

V219 V220 V221 V222

V223 V224

V225 V226 V227 V228 V229 V230 V231 V232 V233 V234 V235 V236 V237 V238_1

V238_2 V239 V240 V241 V242

V243 V244 V245 V246 V247 V248 V249 V250 V251 V252 V253 V254 V255 V256 V257

V258 V259 V260

V261 V262 V263 V264 V265 V266 V267 V268 V269 V270 V271 V272 V273 V274 V275

V276 V277 V278 V279 V280

V281 V282 V283 V284 V285 V286 V287 V288 V289 V290 V291 V292 V293A

V294 V295 V296 V297 V298 V299 V300 V301 V302 V303 V304 V305 V306 V307 V308

V309 V310 V311

V312A V313 V314 V317

V318 V319 V320 V321 V322 V323 V324 V325 V326 V327

SEST1 SEST2 SEST3 SEST4 SEST5 SEST6 SEST7

V335 V336 V337 V338 V339 V340 V341 V342 V343 V344 V345 V346 V347 V348 V349 V350

V351 V352 V353 V354 V355 V356A V356B V356C V356D

V356E V356F V356G V356H V356I V356J V360A V360B V362A V363 V365 V367 V369A

V369B V370B FENOTIPO

CIUDAD PAIS SEXO EDAD ESTUDIOS HIJOS PAREJA SITUACION;

MISSING ARE ALL (99);

USEVARIABLES ARE PWB1 PWB7 PWB17 PWB24

PWB8 PWB12 PWB22 PWB25

PWB4 PWB18 PWB10 PWB14 PWB29 PWB21 PWB27 PWB28

PWB6 PWB11 PWB15 PWB16

PWB20

PDISC1 PDISC2 PDISC3 PDISC5

SEST1 SEST2 SEST3 SEST5;

CATEGORICAL ARE PWB1 PWB7 PWB17 PWB24

PWB8 PWB12 PWB22 PWB25

PWB4 PWB18 PWB10 PWB14 PWB29 PWB21 PWB27 PWB28

PWB6 PWB11 PWB15 PWB16

PWB20

PDISC1 PDISC2 PDISC3 PDISC5

SEST1 SEST2 SEST3 SEST5;

ANALYSIS:

ESTIMATOR IS WLSMV;

ITERATIONS = 1000;

CONVERGENCE = 0.005;

COVERAGE = 0.10;

BOOTSTRAP=1000;

MODEL:

PDISC BY PDISC1 PDISC2 PDISC3 PDISC5;

SEST BY SEST1 SEST2 SEST3 SEST5;

PWB_SA BY PWB1 PWB7 PWB17 PWB24;

PWB_PR BY PWB8 PWB12 PWB22 PWB25;

PWB_AU BY PWB4 PWB18;

PWB_DE BY PWB10 PWB14 PWB29;

PWB_PG BY PWB21 PWB27 PWB28;

PWB_PO BY PWB6 PWB11 PWB15 PWB16 PWB20;

SEST ON PDISC;

PWB_SA ON PDISC;

PWB_PR ON PDISC;

PWB_AU ON PDISC;

PWB_DE ON PDISC;

PWB_PG ON PDISC;

PWB_PO ON PDISC;

PWB_SA ON SEST;

PWB_PR ON SEST;

PWB_AU ON SEST;

PWB_DE ON SEST;

PWB_PG ON SEST;

PWB_PO ON SEST;

MODEL INDIRECT:

PWB_SA IND PDISC;

PWB_PR IND PDISC;

PWB_AU IND PDISC;

PWB_DE IND PDISC;

PWB_PG IND PDISC;

PWB_PO IND PDISC;

OUTPUT:

SAMPSTAT RESIDUAL STANDARDIZED(STDYX) CINTERVAL(BCBOOTSTRAP);

**SEM_DISCSESTPWBmoder.inp**

DATA:

FILE IS "datitems99.DAT";

FORMAT IS FREE;

TYPE IS INDIVIDUAL;

NOBSERVATIONS ARE 853;

VARIABLE:

NAMES ARE cv1 cv2 cv3 cv4 cv5 cv6 cv7 cv8 cv9 cv10 cv11 cv12 cv13 cv14

cv15 cv16 cv17 cv18 cv19 cv20 cv21 cv22 cv23 cv24 cv25 cv26

sf1 sf2 sf3 sf4 sf5 sf6 sf7 sf8 sf9 sf10 sf11 sf12

GHQ121 GHQ122 GHQ123 GHQ124 GHQ125 GHQ126 GHQ127 GHQ128

GHQ129 GHQ1210 GHQ1211 GHQ1212

di1 di2 di3 di4 di5

keyes1 keyes2 keyes3 keyes4 keyes5 keyes6 keyes7 keyes8 keyes9 keyes10

keyes11 keyes12

keyes13 keyes14 keyes15 keyes16 keyes17 PWB1 PWB2 PWB3 PWB4 PWB5

PWB6 PWB7 PWB8

PWB9 PWB10 PWB11 PWB12 PWB13 PWB14 PWB15 PWB16 PWB17 PWB18

PWB19 PWB20 PWB21

PWB22 PWB23 PWB24 PWB25 PWB26 PWB27 PWB28 PWB29

ruiz1 ruiz2 ruiz3 ruiz4 ruiz5 ruiz6 ruiz7 ruiz8 ruiz9 ruiz10 ruiz11

ruiz12 ruiz13 ruiz14 ruiz15

ruiz16 ruiz17 ruiz18 ruiz19 ruiz20 ruiz21 ruiz22 ruiz23 ruiz24

oq1 oq2 oq3 oq4 oq5 oq6 oq7 oq8 oq9 oq10 oq11 oq12 oq13 oq14 oq15 oq16

oq17 oq18 oq19 oq20

oq21 oq22 oq23 oq24 oq25 oq26 oq27 oq28 oq29 oq30 oq31 oq32 oq33 oq34

oq35 oq36 oq37 oq38 oq39

oq40 oq41 oq42 oq43 oq44 oq45

DSE1 DSE2 DSE3 DSE4 DSE5 DSE6 DSE7 DSE8 DSE9 DSE10

V181 V182

V183 V184 V185 V186

AUTCOL1 AUTCOL2 AUTCOL3 AUTCOL4 AUTCOL5 AUTCOL6

AUTOSIM

V194A V194B V194C

V195A V195B V195C

V196A V196B V196C V196D

V197A V197B V197C V197D V197E V197F

V198A V198B V198C V198D V198E V198F V198G V198H V198I V198J V198K V198L

V199 V200

PDISC1 PDISC2 PDISC3 PDISC4 PDISC5

V206 V207

V208 V209A

V209B V209C V209D V209E V209F V209G V210 V211A1 V211A2 V211B1 V211B2 V211C1

V211C2 V211D1 V211D2

V211E1 V211E2 V211F1 V211F2 V211G1 V211G2 V212 V213 V214 V215 V216 V217 V218

V219 V220 V221 V222

V223 V224

V225 V226 V227 V228 V229 V230 V231 V232 V233 V234 V235 V236 V237 V238_1

V238_2 V239 V240 V241 V242

V243 V244 V245 V246 V247 V248 V249 V250 V251 V252 V253 V254 V255 V256 V257

V258 V259 V260

V261 V262 V263 V264 V265 V266 V267 V268 V269 V270 V271 V272 V273 V274 V275

V276 V277 V278 V279 V280

V281 V282 V283 V284 V285 V286 V287 V288 V289 V290 V291 V292 V293A

V294 V295 V296 V297 V298 V299 V300 V301 V302 V303 V304 V305 V306 V307 V308

V309 V310 V311

V312A V313 V314 V317

V318 V319 V320 V321 V322 V323 V324 V325 V326 V327

SEST1 SEST2 SEST3 SEST4 SEST5 SEST6 SEST7

V335 V336 V337 V338 V339 V340 V341 V342 V343 V344 V345 V346 V347 V348 V349 V350

V351 V352 V353 V354 V355 V356A V356B V356C V356D

V356E V356F V356G V356H V356I V356J V360A V360B V362A V363 V365 V367 V369A

V369B V370B FENOTIPO

CIUDAD PAIS SEXO EDAD ESTUDIOS HIJOS PAREJA SITUACION;

MISSING ARE ALL (99);

USEVARIABLES ARE PWB1 PWB7 PWB17 PWB24

PWB8 PWB12 PWB22 PWB25

PWB4 PWB18 PWB10 PWB14 PWB29 PWB21 PWB27 PWB28

PWB6 PWB11 PWB15 PWB16

PWB20

PDISC1 PDISC2 PDISC3 PDISC5

SEST1 SEST2 SEST3 SEST5;

ANALYSIS:

TYPE= general random;

ESTIMATOR IS ML;

ALGORITHM=INTEGRATION;

MODEL:

PDISC BY PDISC1 PDISC2 PDISC3 PDISC5;

SEST BY SEST1 SEST2 SEST3 SEST5;

SEST@1;

PWB_SA BY PWB1 PWB7 PWB17 PWB24;

PWB_PR BY PWB8 PWB12 PWB22 PWB25;

PWB_AU BY PWB4 PWB18;

PWB_DE BY PWB10 PWB14 PWB29;

PWB_PG BY PWB21 PWB27 PWB28;

PWB_PO BY PWB6 PWB11 PWB15 PWB16 PWB20;

PDISCXSEST | PDISC XWITH SEST;

PWB_SA ON PDISC(b11);

PWB_PR ON PDISC(b12);

PWB_AU ON PDISC(b13);

PWB_DE ON PDISC(b14);

PWB_PG ON PDISC(b15);

PWB_PO ON PDISC(b16);

PWB_SA ON SEST(b21);

PWB_PR ON SEST (b22);

PWB_AU ON SEST (b23);

PWB_DE ON SEST (b24);

PWB_PG ON SEST (b25);

PWB_PO ON SEST (b26);

PWB_SA ON PDISCXSEST(b31);

PWB_PR ON PDISCXSEST(b32);

PWB_AU ON PDISCXSEST(b33);

PWB_DE ON PDISCXSEST(b34);

PWB_PG ON PDISCXSEST(b35);

PWB_PO ON PDISCXSEST(b36);

MODEL CONSTRAINT:

NEW(LOW_W MED_W HIGH_W SIMP_LO1 SIMP_MED1 SIMP_HI1);

NEW(SIMP_LO2 SIMP_MED2 SIMP_HI2 SIMP_LO3 SIMP_MED3 SIMP_HI3);

NEW(SIMP_LO4 SIMP_MED4 SIMP_HI4 SIMP_LO5 SIMP_MED5 SIMP_HI5);

NEW(SIMP_LO6 SIMP_MED6 SIMP_HI6);

LOW_W = -1; ! -1 SD below mean of W

MED_W = 0; ! mean of W

HIGH_W = 1; ! +1 SD below mean of W

SIMP_LO1 = b11 + b31*LOW_W;

SIMP_MED1 = b11 + b31*MED_W;

SIMP_HI1 = b11 + b31*HIGH_W;

SIMP_LO2 = b12 + b32*LOW_W;

SIMP_MED2 = b12 + b32*MED_W;

SIMP_HI2 = b12 + b32*HIGH_W;

SIMP_LO3 = b13 + b33*LOW_W;

SIMP_MED3 = b13 + b33*MED_W;

SIMP_HI3 = b13 + b33*HIGH_W;

SIMP_LO4 = b14 + b34*LOW_W;

SIMP_MED4= b14 + b34*MED_W;

SIMP_HI4= b14 + b34*HIGH_W;

SIMP_LO5 = b15 + b35*LOW_W;

SIMP_MED5 = b15 + b35*MED_W;

SIMP_HI5 = b15 + b35*HIGH_W;

SIMP_LO6 = b16 + b36*LOW_W;

SIMP_MED6= b16 + b36*MED_W;

SIMP_HI6= b16 + b36*HIGH_W;

PLOT(LOMOD5 MEDMOD5 HIMOD5);

LOOP(XVAL,-3,3,0.1);

!LOMOD1 = (b11 + b31*LOW_W)*XVAL;

!MEDMOD1 = (b11 + b31*MED_W)*XVAL;

!HIMOD1 = (b11 + b31*HIGH_W)*XVAL;

!LOMOD2 = (b12 + b32*LOW_W)*XVAL;

!MEDMOD2 = (b12 + b32*MED_W)*XVAL;

!HIMOD2 = (b12 + b32*HIGH_W)*XVAL;

!LOMOD3 = (b13 + b33*LOW_W)*XVAL;

!MEDMOD3 = (b13 + b33*MED_W)*XVAL;

!HIMOD3 = (b13 + b33*HIGH_W)*XVAL;

!LOMOD4 = (b14 + b34*LOW_W)*XVAL;

!MEDMOD4 = (b14 + b34*MED_W)*XVAL;

!HIMOD4 = (b14 + b34*HIGH_W)*XVAL;

LOMOD5 = (b15 + b35*LOW_W)*XVAL;

MEDMOD5 = (b15 + b35*MED_W)*XVAL;

HIMOD5 = (b15 + b35*HIGH_W)*XVAL;

!LOMOD6 = (b16 + b36*LOW_W)*XVAL;

!MEDMOD6 = (b16 + b36*MED_W)*XVAL;

!HIMOD6 = (b16 + b36*HIGH_W)*XVAL;

PLOT:

TYPE = plot2;

OUTPUT:

SAMPSTAT RESIDUAL STANDARDIZED(STDYX);

**SEM_SESTPWB.inp**

DATA:

FILE IS "datitems99.DAT";

FORMAT IS FREE;

TYPE IS INDIVIDUAL;

NOBSERVATIONS ARE 853;

VARIABLE:

NAMES ARE cv1 cv2 cv3 cv4 cv5 cv6 cv7 cv8 cv9 cv10 cv11 cv12 cv13 cv14

cv15 cv16 cv17 cv18 cv19 cv20 cv21 cv22 cv23 cv24 cv25 cv26

sf1 sf2 sf3 sf4 sf5 sf6 sf7 sf8 sf9 sf10 sf11 sf12

GHQ121 GHQ122 GHQ123 GHQ124 GHQ125 GHQ126 GHQ127 GHQ128

GHQ129 GHQ1210 GHQ1211 GHQ1212

di1 di2 di3 di4 di5

keyes1 keyes2 keyes3 keyes4 keyes5 keyes6 keyes7 keyes8 keyes9 keyes10

keyes11 keyes12

keyes13 keyes14 keyes15 keyes16 keyes17 PWB1 PWB2 PWB3 PWB4 PWB5

PWB6 PWB7 PWB8

PWB9 PWB10 PWB11 PWB12 PWB13 PWB14 PWB15 PWB16 PWB17 PWB18

PWB19 PWB20 PWB21

PWB22 PWB23 PWB24 PWB25 PWB26 PWB27 PWB28 PWB29

ruiz1 ruiz2 ruiz3 ruiz4 ruiz5 ruiz6 ruiz7 ruiz8 ruiz9 ruiz10 ruiz11

ruiz12 ruiz13 ruiz14 ruiz15

ruiz16 ruiz17 ruiz18 ruiz19 ruiz20 ruiz21 ruiz22 ruiz23 ruiz24

oq1 oq2 oq3 oq4 oq5 oq6 oq7 oq8 oq9 oq10 oq11 oq12 oq13 oq14 oq15 oq16

oq17 oq18 oq19 oq20

oq21 oq22 oq23 oq24 oq25 oq26 oq27 oq28 oq29 oq30 oq31 oq32 oq33 oq34

oq35 oq36 oq37 oq38 oq39

oq40 oq41 oq42 oq43 oq44 oq45

DSE1 DSE2 DSE3 DSE4 DSE5 DSE6 DSE7 DSE8 DSE9 DSE10

V181 V182

V183 V184 V185 V186

AUTCOL1 AUTCOL2 AUTCOL3 AUTCOL4 AUTCOL5 AUTCOL6

AUTOSIM

V194A V194B V194C

V195A V195B V195C

V196A V196B V196C V196D

V197A V197B V197C V197D V197E V197F

V198A V198B V198C V198D V198E V198F V198G V198H V198I V198J V198K V198L

V199 V200

PDISC1 PDISC2 PDISC3 PDISC4 PDISC5

V206 V207

V208 V209A

V209B V209C V209D V209E V209F V209G V210 V211A1 V211A2 V211B1 V211B2 V211C1

V211C2 V211D1 V211D2

V211E1 V211E2 V211F1 V211F2 V211G1 V211G2 V212 V213 V214 V215 V216 V217 V218

V219 V220 V221 V222

V223 V224

V225 V226 V227 V228 V229 V230 V231 V232 V233 V234 V235 V236 V237 V238_1

V238_2 V239 V240 V241 V242

V243 V244 V245 V246 V247 V248 V249 V250 V251 V252 V253 V254 V255 V256 V257

V258 V259 V260

V261 V262 V263 V264 V265 V266 V267 V268 V269 V270 V271 V272 V273 V274 V275

V276 V277 V278 V279 V280

V281 V282 V283 V284 V285 V286 V287 V288 V289 V290 V291 V292 V293A

V294 V295 V296 V297 V298 V299 V300 V301 V302 V303 V304 V305 V306 V307 V308

V309 V310 V311

V312A V313 V314 V317

V318 V319 V320 V321 V322 V323 V324 V325 V326 V327

SEST1 SEST2 SEST3 SEST4 SEST5 SEST6 SEST7

V335 V336 V337 V338 V339 V340 V341 V342 V343 V344 V345 V346 V347 V348 V349 V350

V351 V352 V353 V354 V355 V356A V356B V356C V356D

V356E V356F V356G V356H V356I V356J V360A V360B V362A V363 V365 V367 V369A

V369B V370B FENOTIPO

CIUDAD PAIS SEXO EDAD ESTUDIOS HIJOS PAREJA SITUACION;

MISSING ARE ALL (99);

USEVARIABLES ARE PWB1 PWB4 PWB6 PWB7 PWB8

PWB10 PWB11 PWB12 PWB14 PWB15 PWB16 PWB17 PWB18

PWB20 PWB21

PWB22 PWB24 PWB25 PWB27 PWB28 PWB29

SEST1 SEST2 SEST3 SEST5;

CATEGORICAL ARE PWB1 PWB4 PWB6 PWB7 PWB8

PWB10 PWB11 PWB12 PWB14 PWB15 PWB16 PWB17 PWB18

PWB20 PWB21

PWB22 PWB24 PWB25 PWB27 PWB28 PWB29

SEST1 SEST2 SEST3 SEST5;

ANALYSIS:

ESTIMATOR IS WLSMV;

ITERATIONS = 1000;

CONVERGENCE = 0.005;

COVERAGE = 0.10;

MODEL:

SEST BY SEST1 SEST2 SEST3 SEST5;

PWB_SA BY PWB1 PWB7 PWB17 PWB24;

PWB_PR BY PWB8 PWB12 PWB22 PWB25;

PWB_AU BY PWB4 PWB18;

PWB_DE BY PWB10 PWB14 PWB29;

PWB_PG BY PWB21 PWB27 PWB28;

PWB_PO BY PWB6 PWB11 PWB15 PWB16 PWB20;

OUTPUT:

SAMPSTAT RESIDUAL STANDARDIZED(STDYX);
