# Supplementary material for: The mediating effect of self-esteem on the relationship between perceived discrimination and psychological well-being in immigrants
Source: PLoS One. 2018 Jun 21;13(6):e0198413. doi: 10.1371/journal.pone.0198413 (PMC6013095; doi:10.1371/journal.pone.0198413)
Supplement: S2 Data — (DOCX) [file pone.0198413.s002.docx]

- 1. Raw data file used to conduce the analyses, in free format: <https://1drv.ms/u/s!Au5ZByp1rtTji7Vl01M0IYKQwm3AZQ>
